# Geographical Distribution and Pattern of Pesticides in Danish Drinking Water 2002–2018: Reducing Data Complexity

**DOI:** 10.3390/ijerph19020823

**Published:** 2022-01-12

**Authors:** Carina Skaarup, Kirstine Wodschow, Denitza D. Voutchkova, Jörg Schullehner, Ole Raaschou-Nielsen, Helle Raun Andersen, Birgitte Hansen, Annette Kjær Ersbøll

**Affiliations:** 1 National Institute of Public Health, University of Southern Denmark, 1455 Copenhagen, Denmark; ikwo@sdu.dk (K.W.); ake@sdu.dk (A.K.E.); 2 Geological Survey of Denmark and Greenland (GEUS), C.F. Møllers Allé 8, 8000 Aarhus, Denmark; dv@geus.dk (D.D.V.); jsc@geus.dk (J.S.); bgh@geus.dk (B.H.); 3 Department of Public Health, Research Unit for Environment, Work and Health, Aarhus University, Bartholins Allé 2, 8000 Aarhus, Denmark; 4 Danish Cancer Society Research Center, Strandboulevarden 49, 2100 Copenhagen, Denmark; ole@cancer.dk; 5 Department of Environmental Science, Aarhus University, Frederiksborgvej 399, 4000 Roskilde, Denmark; 6 Department of Public Health, Clinical Pharmacology, Pharmacy and Envinronmental Medicine, University of Southern Denmark, J.B. Winsløws Vej 17A, 5000 Odense, Denmark; hrandersen@health.sdu.dk

**Keywords:** pesticides, drinking water, monitoring pesticides, drinking water quality, geographic distribution of pesticides

## Abstract

Pesticides are a large and heterogenous group of chemicals with a complex geographic distribution in the environment. The purpose of this study was to explore the geographic distribution of pesticides in Danish drinking water and identify potential patterns in the grouping of pesticides. Our data included 899,169 analyses of 167 pesticides and metabolites, of which 55 were identified above the detection limit. Pesticide patterns were defined by (1) pesticide groups based on chemical structure and pesticide–metabolite relations and (2) an exploratory factor analysis identifying underlying patterns of related pesticides within waterworks. The geographic distribution was evaluated by mapping the pesticide categories for groups and factor components, namely those detected, quantified, above quality standards, and not analysed. We identified five and seven factor components for the periods 2002–2011 and 2012–2018, respectively. In total, 16 pesticide groups were identified, of which six were representative in space and time with regards to the number of waterworks and analyses, namely benzothiazinone, benzonitriles, organophosphates, phenoxy herbicides, triazines, and triazinones. Pesticide mapping identified areas where multiple pesticides were detected, indicating areas with a higher pesticide burden. The results contribute to a better understanding of the pesticide pattern in Danish drinking water and may contribute to exposure assessments for future epidemiological studies.

## 1. Introduction

In many places around the world, ground water and surface water are important sources for drinking water production and are vulnerable to pesticide contamination. The potential health risks from contaminated drinking water are of increasing interest and a major concern in the population. Pesticides are chemicals with the main purpose of controlling pests, including fungi, weeds, and insects, with the correspondent pesticides characterised as fungicides, herbicides, and insecticides, respectively [1]. Pesticides are applied on conventional agricultural fields, park areas, greenhouses, sport facilities, and similar areas. Pesticides are special, as they are a group of known hazardous chemicals that are deliberately spread in nature, with potentially harmful effects on other organisms [2].

As a result of the multiple target organisms and purpose of application, the term pesticide describes a large and heterogenous group of chemicals and their metabolites [1]. Currently, 456 pesticides are approved for use in the European Union (EU). In total, the EU pesticide database includes 1456 pesticides classified into 21 different groups [3].

In the EU, the approval and use of pesticides are regulated by the European Commission (EC) and the national Environmental Protection Agencies of the member states. The European Food Safety Agency (EFSA) is responsible for the peer review of the risk assessments of the active substances used in pesticides in the EU. To ensure the sustainable use of pesticides, the EU legislation requires pesticides to be effective and have no harmful effects with regards to human and animal health and impacts on the environment. All pesticide products (active ingredients) are thoroughly assessed upon approval [4].

The EU drinking water directive [5] defines the maximum concentration allowed in drinking water as 0.1 µg/L for a single pesticide or metabolite, known as the drinking water quality standard (DWQS), and 0.5 µg/L for the total sum of pesticides (DWQS-sum). The drinking water directive was implemented in Danish law as Drikkevandsbekendtgørelsen [6], including a list of pesticides for mandatory surveillance. Many of the pesticides included in Drikkevandsbekendtgørelsen and the pesticides detected in Danish drinking water are no longer approved in Denmark (Table S1, Supplementary Material).

The potential health impact of pesticide exposure at low concentrations in the general population is difficult to investigate because exposure differences are small and the population is exposed to complex mixtures of pesticides from different sources, e.g., the ingestion of residues in food products and drinking water or residential proximity to agricultural areas. Thus, residential distance to agricultural use of pesticides and crop density around the residences has been used as a proxy for pesticide exposure and found to be associated with increased risk of childhood leukaemia [7,8]. Further, studies have identified an increased risk of childhood leukaemia when the mother is exposed to pesticides at work or from use in the home during pregnancy [9]. Additionally, organic food consumption has been associated with reduced risk of cancer [10], although the potential impact of pesticides in drinking water on cancer risk has only been scarcely investigated. Because of the low permissible concentrations of pesticides in drinking water, exposure from drinking water is low compared to exposure levels from residues in food items. However, the mixture of pesticides in drinking water differs from those in food items, and for some health outcomes such as cancer, there might not be a safe threshold for exposure.

The International Agency for Research on Cancer (IARC) has published a list of classifications for carcinogenic agents [11]. The pesticides diazinon, glyphosate, lindane, malathion, and pentachlorophenol are classified as level 2A (probably carcinogenic to humans), while 2,4-D, 2,4,6-trichlorophenol, DDT, hexachlorobenzene, parathion, and tetrachlorvinphos are classified as level 2B (possibly carcinogenic) [11,12].

The highly decentralised Danish water supply relies entirely on ground water and consists of local waterworks linked to water supply areas (WSA), supplying the majority of Danish households with drinking water. Water treatment is limited and usually only consists of aeration and sand filtration.

Regular sampling and analyses are performed on drinking water sampled at the waterworks outlet to monitor water quality. Sampling frequency can vary from once every three years to multiple times a year, depending on the water production volume of the waterworks. Water quality, including pesticide contamination, is reported to the Danish national well database Jupiter [13].

Voutchkova et al. (2021) have recently studied pesticides in Danish drinking water over the period 2002–2019. They found that the drinking water had been analysed for 449 different pesticides and metabolites within the period, and as a result of changes in the monitoring program and screenings, the pesticide dataset is highly heterogenous. The frequency and total number of analyses for each pesticide and waterworks varied a lot (based on Jupiter database). The sampling and analysis pattern resulted in a heterogeneous data structure, including partial data series for a large number of included pesticides [14]. Although pesticides in Danish drinking water have been quantified, further research is needed to better understand the distribution patterns of the numerous pesticides, which may contribute to better exposure assessment in future epidemiological studies of associations between pesticides and health.

The aim of our study, therefore, was to explore the geographical distribution of pesticides in drinking water in Denmark and to identify potential patterns of co-occurrence of pesticides. This included the identification of pesticide groups in relation to the chemical structure and geographic pattern of detected pesticides.

## 2. Materials and Methods

### 2.1. Data Sources

Data on pesticide concentrations in Danish waterworks were extracted from the Jupiter database on 5 May 2020. Pesticide concentrations measured in the period 2002–2018 were included in the study. A detailed description of the data structure and collection is given by Voutchkova et al. (2021). Briefly, the extracted data included all reported chemical analyses of pesticides, their degradation products, and related substances. The initial data management process included pre-processing and quality control to exclude non-trusted observations and samples not relevant for the study. For further information, see the study by Voutchkova et al. (2021).

### 2.2. Public Water Supply

Information on public waterworks and the geographic coordinates were obtained from the Jupiter database [13]. Information on locations of WSAs and links to waterworks were defined by Schullehner and Hansen (2014) and modified by Wodschow et al. (2018) [15,16].

### 2.3. Data Management

**Figure 1:**
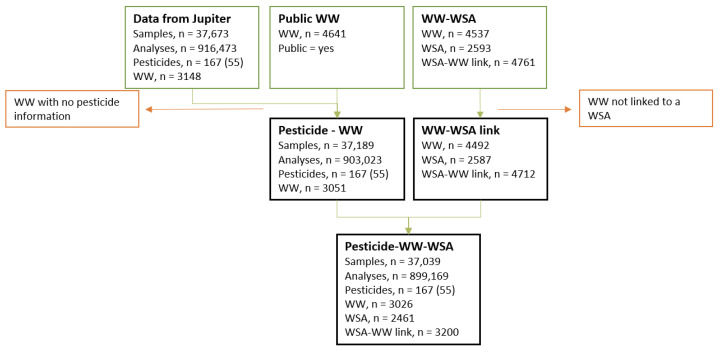
Flow chart illustrating the different data sources and exclusion criteria. Water samples and laboratory analyses are presented by number of observations. Pesticides are represented by the number of pesticides in the dataset, with the number of pesticides above LOD in brackets. Abbreviations: WW: waterworks; WSA: water supply area; LOD: limit of detection.

Pesticide data were linked to waterworks using a waterworks ID. Waterworks with missing data on geographic coordinates or address were excluded from the study. Next, the pesticide data were linked to WSAs via spatial joints between waterworks and WSAs. Waterworks not linked to a WSA were excluded. The process is illustrated in Figure 1.

Based on measured pesticide concentrations in waterworks, pesticides were categorised into 5 categories: <0.010 µg/L (pesticide not detected), 0.010–0.029 µg/L (pesticide detected, not quantified), 0.030–0.10 µg/L (quantified), >0.10 µg/L (exceeding drinking water quality standard), and no data/not analysed.

A yearly pesticide category was defined first at the waterworks level and subsequently at the WSA level. The category was defined both for each pesticide and as an overall pesticide category, including all pesticides analysed. The mean of measurements for each specific pesticide was computed across samples and used to define the pesticide category. Similar, the DWQS-sum criteria were evaluated at the sample level.

For waterworks with multiple samples per year, the category was defined by the maximum measured concentration for each pesticide. The overall pesticide category was defined as the maximum category observed for any pesticide at the waterworks. Similar, the WSA category was defined for each pesticide based on the waterworks category. WSAs linked with multiple waterworks were categorised by the maximum concentration from the relevant waterworks. The overall pesticide category was defined as the maximum category observed.

### 2.4. Data Exploration

The data exploration included an overview of detected pesticides, the geographical variation in groups of pesticides with similar chemical structures, and a factor analysis. For the following analyses, data were restricted, and only pesticides observed at or above the limit of detection (≥LOD) at least once were included. Pesticides included in the study and information on the analysis status are given in Table S1 (Supplementary Material).

#### 2.4.1. Pesticide Groups

All pesticides were assigned a pesticide group. A group was defined based on the chemical structure of the pesticide, as defined by the Compendium of Pesticide Common Names (http://alanwood.net/pesticides/index.html, accessed on 22 July 2021), and metabolites were assigned to the group of the parent pesticides. Each group was evaluated by the prevalence of each pesticide group in terms of numbers of waterworks and WSAs where analyses were performed, analyses across the full study period, and detection of pesticides.

The geographic distribution and pattern of pesticide groups in Denmark were analysed at the WSA level. For each WSA, the maximum category measured for any of the pesticides in the pesticide group defined the pesticide category. The distribution of pesticide groups was further analysed for potential variation over time. To account for the sampling structure and to assure a minimum of one sampling event for all waterworks, the following time periods were defined: 2002–2005, 2006–2008, 2009–2011, 2012–2015, and 2016–2018.

#### 2.4.2. Factor Analysis

We used an exploratory factor analysis (EFA) [17] to identify underlying structures and correlations between pesticides and to reduce data dimensions, resulting in a set of new variables called factor components. Pesticide concentrations were computed at the waterworks level for all analysed pesticides. Observations below LOD were assigned the concentration at LOD/2 [18], and missing observations were substituted with −1. Hornung and Reed (1990) suggest two methods for handling observations below the detection limit: LOD/2 or LOD/√2. If more than half of the observations are nondetectable, assigning the value LOD/2 is recommended. As that was the case in this dataset, we used LOD/2.

Two criteria were set for a waterworks and a pesticide to be included in the factor analysis: (1) a minimum of 10 different pesticides were analysed at each waterworks and (2) a pesticide had been analysed at a minimum of 10% of the included waterworks.

The sampling adequacy of the dataset (eligibility for factor analysis) was assessed based on a Keyser–Meyer–Olvin (MSA) score > 0.6 (60%) [17].

When interpreting the result, we set a criterion for factor-variable loadings being >0.45 or <−0.45, hence only variables with strong loadings were used. Further, we applied an oblique (i.e., non-orthogonal) factor rotation (with promax), assuming the pesticides were correlated and allowed correlation between factor components, to assure optimum fit. The analysis was performed using SAS 9.4 PROC FACTOR [19].

The selection of the optimum number of factor components is a choice relating to how much of the variation in data can be explained by the selected factor components. A set of objective criteria were used, including an evaluation of eigenvalues (amount of variance explained by the factor component) with a cut-off value of 1, a scree plot (changes in eigenvalues as the number of factors increase), and Horn’s parallel analysis (use of simulated data to define factor cut-off) [17]. The eigenvalue criteria are set at 1; hence, factor component with eigenvalue ≥ 1 are retained. The scree plot evaluates the relation between explained variance and number of components. The optimum number of components is selected based on the shape of the curve, where an elbow shape indicates the cut-off of number of factors to retain. Further, the Horn’s parallel analysis calculates eigenvalues for a simulated dataset (with the same numbers of observations and variables as the dataset of interest). The number of factors where the simulated eigenvalue is higher than the eigenvalue for the dataset defines the cut-off for factors to be retained. When plotted, the number of components at which the scree plot and parallel analysis intersect indicates the number of factor components to retain.

The evaluation was performed using the following steps:

(1) Perform factor analysis with the specifications listed above;
(2) Evaluate MSA, eigenvalues, and scree plot, including Horn’s parallel analysis, and select optimum number of factor components;
(3) Perform factor analysis, specifying number of components and rotation;
(4) Evaluate factor pattern—if pesticides are observed with poor loadings (i.e., −0.45 to 0.45) to the factor components (not reaching the pre-set criteria for inclusion), the pesticides are removed and steps 1–4 are repeated.

When all pesticides meet the criteria of a strong loading score to one or more factor components, the analysis is completed and the factor pattern is presented.

#### 2.4.3. Sensitivity Analysis

The main factor analysis was performed for the maximum measured concentration, and the missing values were substituted with −1. To evaluate whether these decisions influenced the factor pattern, we performed the following sensitivity analyses: (1) the pesticide concentration was calculated as the mean concentration measured at the waterworks for the period; (2) the pesticide concentration was categorised (0/1) based on LOQ (≥0.030 µg/L); (3) the pesticide concentration was categorised (0/1) based on DWQS (>0.10 µg/L). All sensitivity analyses were performed with datasets where the missing observations were substituted with either 0 or −1.

## 3. Results

### 3.1. Descriptive Results

The dataset was composed of 899,169 analyses from 3026 waterworks linked to 2461 WSAs, with analyses for 167 different pesticides and metabolites. Of these, 55 were detected in 9740 analyses (1.08% of the total dataset) at 1281 waterworks in 1157 WSAs. Table S1 (Supplementary Material) presents the pesticides and metabolites included in the study. To summarise, the dataset included 25 pesticides and 30 metabolites, of which 44 were classified as herbicides (Hrb), six as fungicides (Fun), and three as insecticides (Ins). Of the 25 pesticides, 22 were restricted or no longer approved in Denmark or EU.

**Figure 2:**
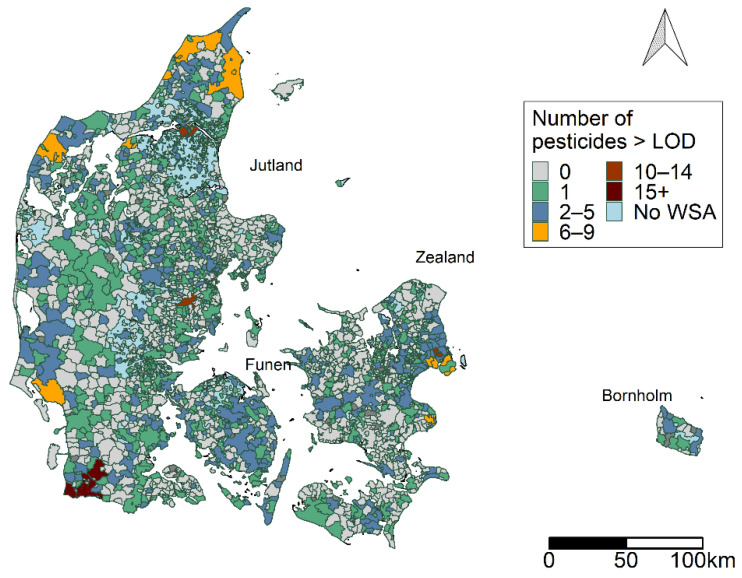
Locations of water supply areas (WSAs) in Denmark, classified by the number of pesticides detected at each WSA above the detection level (LOD) for the period 2002–2018. Map contains data from the Danish Agency for Data Supply and Efficiency, municipality borders, 2019.

The locations of WSAs are shown in Figure 2. The number of different pesticides detected above the LOD (>0.01 µg/L) is illustrated by the colour of the WSA.

The map in Figure 2 shows the numbers of different pesticides and metabolites detected in the WSAs. Areas were found across the country with low number of pesticides detected, while other areas in the north, west, and south of Jutland had higher numbers of pesticides. A similar pattern was observed in the mid-east of Jutland and Zealand, where the areas around the capital region showed a higher pesticide burden. In total, 953 (38.7%) of the WSAs contained detected pesticides.

### 3.2. Pesticide Groups

**Figure 3:**
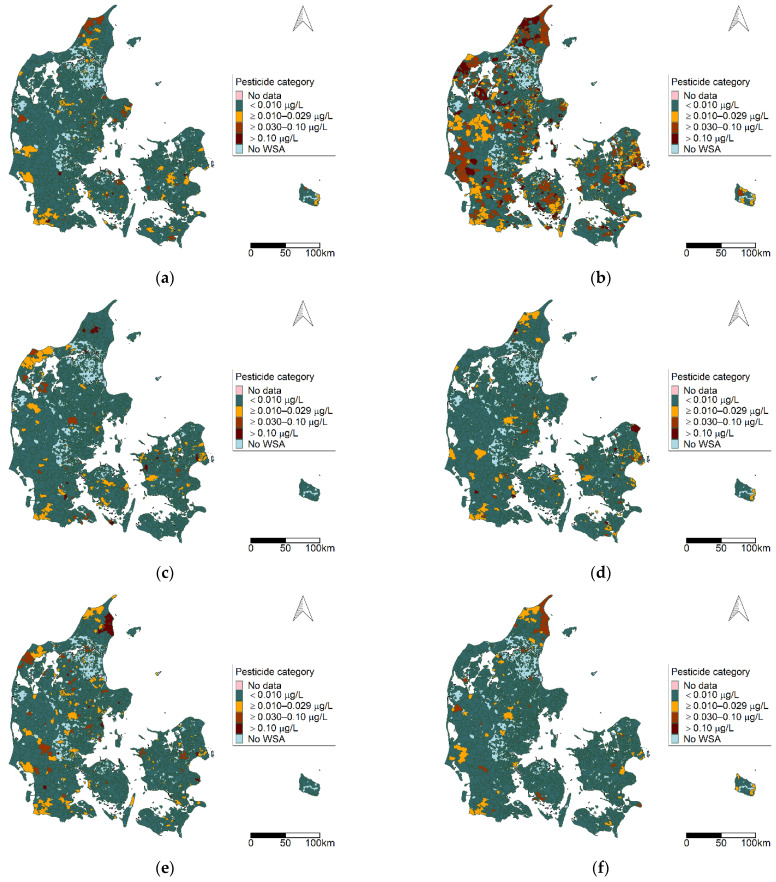
Geographic distribution of pesticide categories at the water supply area (WSA) level for selected pesticide groups for the period 2002–2018: (**a**) benzothiazinones; (**b**) benzonitriles; (**c**) organophosphates; (**d**) phenoxy herbicides; (**e**) triazines; (**f**) triazinones. Map contains data from the Danish Agency for Data Supply and Efficiency, municipality borders, 2019.

The 55 pesticides included in the dataset were assigned to 16 pesticide groups (see Table S1, Supplementary Material). Figure 3a–f presents six pesticide groups of interest.

From Figure 3a–f, one can observe that each pesticide group has a unique distribution pattern, and that all parts of Denmark are represented. Benzonotriles (group b) were the most prevalent group detected of the groups presented. In addition, we observed areas where multiple pesticide groups were detected, which included the northern and north-western part of Jutland, the southwestern and central part of Jutland, and eastern parts of Zealand and Bornholm.

#### Variation over Time

The geographical distribution of pesticide groups was evaluated over five periods: 2002–2005, 2006–2008, 2009–2011, 2012–2015, and 2016–2018. No or limited variation over time was observed for benzothiazinones (detected at 51–60 WSAs) and triazinones (detected at 20–41 WSAs). A reduction in the number of areas with detection of phenoxy herbicides was observed in 2002–2005 and 2006–2008 (at 61 and 66 WSAs respectively) compared to after 2009 (at 46, 36, and 34, respectively).

A gradual decrease was observed for the benzonitriles throughout the period—from 514 WSAs in 2002–2005 to 298 WSAs in 2016–2018. An increase in the number of areas with the detection of organophosphates was seen in 2012–2015 and 2016–2018 (at 67 and 48 WSAs, respectively) as compared to before 2012 (detected at 4, 1, and 19 WSAs, respectively). More sporadic variation was observed for the triazines, detected in 51 to 105 WSAs with increases from 2006–2008 to 2012–2015 (at 51, 63, and 92 WSAs, respectively). The variation in pesticide categories over time within pesticide groups is given in the Supplementary Materials, Figures S1a–f, S2a–f, S3a–f, S4a–f, S5a–f, S6a–f and S7a–f.

### 3.3. Factor Analysis

Based on initial data inspection, the dataset was split into two periods (2002–2011 and 2012–2018) based on changes in the analysis frequency and an increase in number of pesticides analysed in the second period. The results from the two periods are presented below.

#### 3.3.1. Factor Analysis 2002–2011

**Figure 4:**
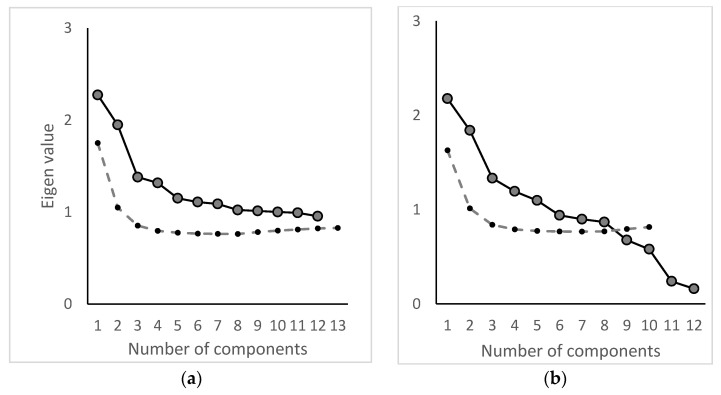
(**a**) Initial scree plot, where six factor components were retained. (**b**) Final scree plot, where five factor components were retained. The dotted line represents Horn’s parallel analysis.

The final dataset for the factor analysis in the study period 2002–2011 was composed of 2935 waterworks and 23 pesticides. In Figure 4a, the scree plot from the initial analysis step is illustrated. Based on the scree plot and the eigenvalue criteria, six factor components were retained. Three iterations of the analysis process were made, and based on the final scree plot (Figure 4b) a total of five factor components were retained based on an eigenvalue > 1, the scree plot, and Horns parallel analysis. The resulting factor pattern is given in Table 1.

The factor pattern given in Table 1 shows the relation between pesticides and factor components given by loading score. The factor pattern, i.e., the pesticide and factor relation—is an indication of the correlation between the pesticides.

Factor 1 is composed of two pesticides (MCPA and dichlorprop), both characterised as phenoxyacid herbicides banned for use in 1996. Factors 2 and 3 represent pesticides of the triazine herbicides group. Factor 2 is composed of the pesticide atrazine and two of its metabolites, while factor 3 is composed of simazine and another atrazine metabolites. Atrazine was banned in 1994, while simazine was banned in 2005. Based on the information from Table 1, no obvious link was identified for the pesticides in factors 4 and 5.

**Figure 5:**
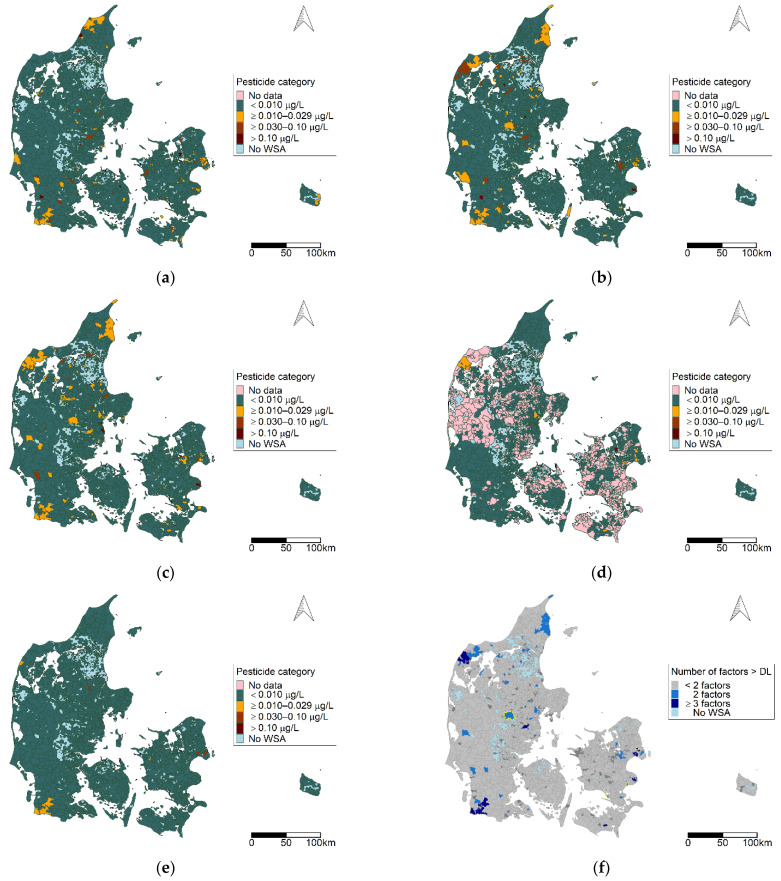
Geographical distribution of factor components 1–5 (**a**–**e**) for 2002–2011, illustrating the pesticide category for pesticides representing the given factor. (**f**) The WSA where multiple factor components were identified. Note that one WSA can represent multiple waterworks, and shows the maximum category represented by the WSA. Abbreviations: WSA: water supply area. Map contains data from the Danish Agency for Data Supply and Efficiency, municipality borders, 2019.

The geographical distribution of factors identified for the study period 2002–2011 is given in Figure 5a–e. Each map represents a corresponding factor component. WSAs where two or more factors were detected are given in Figure 5f. Hydroxyatrazine represents both factor 2 and 3 and defines the factor status in 9 WSAs (indicated by a yellow border). Areas where the specific pesticide defines the factor status are highlighted (see Supplementary Material Figure S8).

#### 3.3.2. Factor Analysis 2012–2018

**Figure 6:**
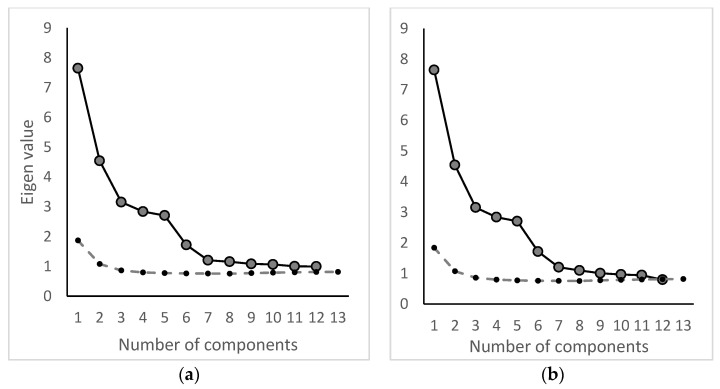
(**a**) Initial scree plot, where seven factor components were retained. (**b**) Final scree plot, where seven factor components were retained. The dotted line represents Horn’s parallel analysis.

The final dataset for the factor analysis for the study period 2012–2018 was composed of 2528 waterworks and 37 pesticides. In Figure 6a, the scree plot from the initial analysis step is illustrated. Based on the scree plot and eigenvalue criteria, seven factor components were retained. In total, two iterations of the analysis process were made, and based on the final scree plot (Figure 6b) a total of seven factor components were retained based on an eigenvalue > 1, the scree plot, and Horn’s parallel analysis. The resulting factor pattern is given in Table 2.

The factor pattern given in Table 2 shows the relation between pesticides and factor components given by the loading score. The factor pattern, i.e., the pesticide and factor relation, is an indication of the correlation between the pesticides.

Factor 1 is composed of pesticides and metabolites of different types and uses.

Factor 2 is composed of pesticides and metabolites used when growing fruit and conifer trees (diuron) and pesticides used when growing potatoes (metribuzin and metalaxyl metabolites).

Factor 3 is a mix of chloridazon and metabolites, pyridazinone herbicides previously used in the production of beets, an azol fungicide, and a phenylsulfamide fungicide, of which DMS is known for use in fruit production.

Factor 4 is composed of atrazine metabolites and the herbicide 2,6-dichlorobenzoic acid used in the production of fruit. Atrazine is used by the Danish State Railways (DSB), in the production of conifer trees, and in plantations.

Factor 5 is composed of phenoxy herbicides used for cereal, grass areas, and garden centres.

Factor 6 is composed of atrazine, metabolites, and hexazinone. These pesticides were previously used in the production of conifer trees, garden centres, and by the DSB.

Factor 7 is composed of an atrazine metabolite and a dithiocarbamate fungicide metabolite.

**Figure 7:**
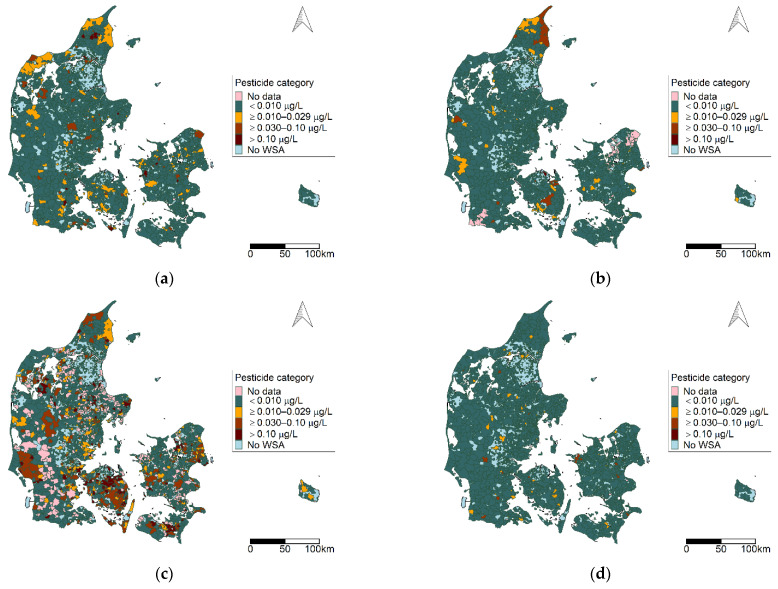

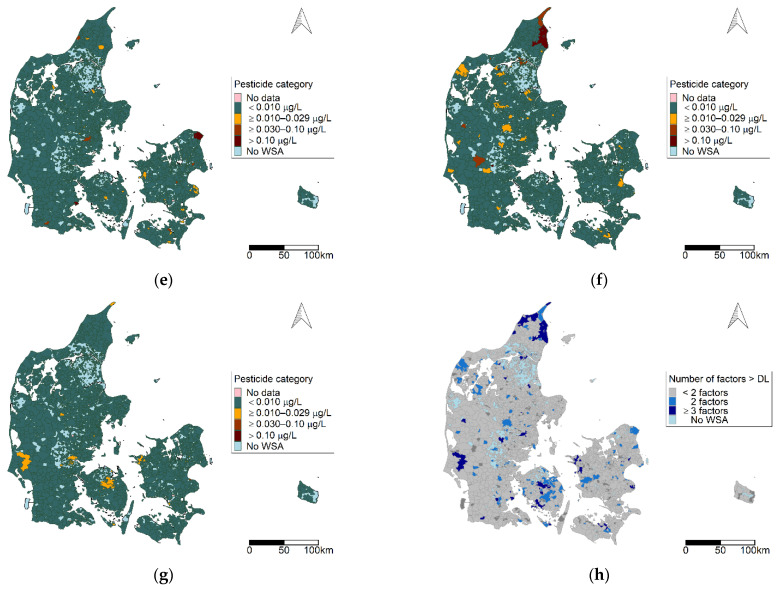
Geographic distribution of factor components, 2012–2018: (**a**–**g**) the geographic pattern of factors 1–7; (**h**) the number of factors represented at each WSA. Note that one WSA can represent multiple waterworks and shows the maximum category represented by the WSA. Abbreviations: WSA: water supply area. Map contains data from the Danish Agency for Data Supply and Efficiency, municipality borders, 2019.

The geographical distribution of factors identified for the study period 2012–2018 is given in Figure 7a–g. Each map represents the corresponding factor component. WSAs where two or more factors were detected are given Figure 7h.

#### 3.3.3. Sensitivity Analysis

The sensitivity analyses using the mean pesticide concentration and substitution of missing values with 0 resulted in similar factor patterns for both 2002–2011 and 2012–2018 (see Supplementary Material Tables S2–S7 and Figures S9–S14).

The sensitivity analysis for the categorised pesticide levels resulted in a singular correlation matrix, meaning it was not applicable for factor analysis.

### 3.4. Compare Patterns

Comparing the results of the factor analysis, factors 1–4 for 2002–2011 were all represented in the 2012–2018 factor pattern. No pesticides from factor 5 from 2002–2011 were represented in the 2012–2018 factor pattern, an expected observation, as all these pesticides were no longer part of the national monitoring program after 2011 or 2014 (2,4-D).

In addition, 23 out of the 32 pesticides observed in the factor pattern for 2012–2018 were first included in the surveillance program in 2012 or later.

The areas observed with a high pesticide burden with regards to multiple pesticide groups and multiple factor components observed in the WSAs (Figure 3, Figure 5f and Figure 7h) reflect the pattern given in Figure 2 with regards to the number of different pesticides observed in a WSA.

## 4. Discussion

### 4.1. Summary of Findings

We identified groups of pesticides in the Danish drinking water based on classification by chemical structure. Our analysis showed that each pesticide group had a unique geographical pattern of detection. It was also seen that multiple pesticide groups overlap and were represented in the same areas, indicating areas with a higher pesticide burden in drinking water.

The factor analyses identified five factor components for the period 2002–2011 and seven factor components for the period 2012–2018. Factors composed or partially composed of the same pesticides were observed for the two periods, reflecting some continuity in data composition and variance. In opposition to this, a number of factors and pesticides that were observed in the 2012–2018 analysis were not included in the 2002–2011 analysis, reflecting the changes in pesticides analysed across time. The factor components had a geographic distribution across the country, with a specific pattern observed for each of the factors.

Comparing the spatial patterns of pesticides groups and factor components, we observed areas where pesticide groups and factors were frequently represented, having a higher pesticide burden.

### 4.2. Comparison with Similar Studies

The majority of studies that have examined patterns of pesticides in water were based on surface water, rivers, or ground water sources [20,21,22,23,24,25,26], while studies involving analyses of pesticides in treated drinking water are sparse [14,27,28,29].

In the review by Syafrudin et al. (2021), the route and potential consequences of pesticides contamination in drinking water sources were described with a focus on the different types of pesticides most likely to reach surface and ground water. The herbicide atrazine was identified as having high potential to contaminate ground water, as it is highly persistent, while the pesticides cyanazine, methyl parathion, and 2,4-D were less likely to be found in ground water due to having a short half-life, high absorption in soil, and water-soluble structure, respectively [28]. The findings in our study reflect this pattern, whereby atrazine and its metabolites were detected in a higher proportion of WSAs than any of the other mentioned pesticides. Cyanazine and 2,4-D were both detected in Danish drinking water, but never exceeded the DWQS. Methyl parathion has not been reported to Jupiter.

The study by Tröger et al. (2021) analysed the presence of contaminants of emerging concern (CEC) including pesticides in both raw water and drinking water in 11 countries in Europe and Asia [29]. Comparing their findings to our study, the pesticides atrazine and the metabolites atrazine-desethyl and atrazine-desisopropyl, bentazon, chloridazon, cyanazine, diuron, hexazinone, and isoproturon were detected in drinking water samples. Atrazine and its metabolites were most prevalent, as they were detected in the largest proportion of the included countries (8 out of 11).

A study in the Netherlands analysed the contamination of pesticides in surface and ground water, as well as wells used for drinking water, i.e., untreated drinking water [27]. Schipper et al. (2008) identified the pesticides bentazon, BAM, bromacil, dikegulac, diuron, and mecoprop at levels above DWQS (>0.1 µg/L) in the raw water samples.

This pattern is consistent with the findings in our study of drinking water with regards to bentazon, BAM, and mecoprop, which were all found at levels >DWQS, while diuron did not exceeded the LOQ. The pesticide bromacil was not reported to Jupiter in the period 2002–2018, although when it was analysed in 2019, it was not detected (data from Jupiter database). Dikegulac was not reported to Jupiter in the study period.

The study by Voutchkova et al. (2021) analysing samples of Danish drinking water evaluated the pesticide status at the waterworks and address levels, identifying the proportions of waterworks and households potentially exposed to pesticides (above the LOQ) in the periods 2002–2019 and 2015–2019. They found that 56% of Danish households had been exposed to pesticides in the period 2002–2019 (37% at 0.030–0.10 µg/L and 19% exceeding the DWQS) [14]. Our findings extend to this analysis and we identified that 38.7% of WSAs detected pesticides in the period 2002–2018 (>LOD).

Multivariate analyses, such as factor analysis and principal component analysis (PCA), can be applied to identify underling structures and correlations within variables in a dataset, as has been shown in this study.

The study by Liu et al. (2003) applied a factor analysis for assessment of ground water quality in Taiwan. The dataset included measurements of 13 hydrochemical parameters related to quality variations in ground water aquifers. The study resulted in two factor components related to seawater salination and arsenic pollution, respectively. By visualising the geographic distribution of factor scores, areas were identified with high seawater salination contamination and arsenic pollution in areas of ground water over-pumping, as expected [30].

The study by Kim et al. (2017) applied PCA and exploratory factor analysis in order to assess temporal and spatial variations in the water quality of the Nakdong River in Korea. The dataset included 15 quality parameters obtained from 28 stations along the river. The factor analysis resulted in two factor components representing sampling stations downstream and upstream of the river, respectively. Factor 1 was defined by organic pollution, nutrients, and the biological activity of algae in the water, while factor 2 was dominated by nutrients and less influenced by the biological activity of algae. The factor patterns reflected the location of agricultural and industrial lands along the river [31].

The study by Mcleod et al. (2017) applied a PCA to evaluate ground water samples from private and public wells with regards to an exposure assessment for epidemiological studies. The variables included health standards (water contaminants, including arsenic) and aesthetic parameters (ions and minerals). The analysis identified similar patterns for public and private water supplies for health-related and aesthetic variables. The study showed that multivariable analysis was useful when identifying underlying patterns within the variables [32].

Other studies have applied factor analysis to questionnaire data [33,34]. Samanic et al. (2005) used data from the agricultural health study [35], including variables related to pesticide use, method of application, crop type, livestock, medical history, and demographic parameters. The questionnaire obtained by Weissenburger-Moser et al. (2017) included similar variables with a focus on pesticide exposure and the durations of different types of exposures.

The resulting factor components characterised the pattern of exposure to pesticides and identified sub-sets within the data [33,34].

These findings illustrate how multivariate analyses can be useful in identifying underlying structures in a dataset and subsequently identify subgroups and geographic patterns, similar to the findings in our study.

### 4.3. Strengths

The large number of samples (n = 37,039) and countrywide representation is a great advantage of our study. The continuous sampling across time and reporting to a central national database resulted in a dataset that would not be possible to obtain elsewhere. The countrywide representation across the 17-year period enabled us to evaluate the development, both with regards to pesticide contamination in drinking water as well as the geographic pattern of pesticide detection.

The highly decentralised structure in the Danish water supply means that multiple waterworks are sampling and performing quality analyses at different times of the year and with different frequencies [14]. Laboratories involved in drinking water quality control must be accredited to perform the sampling and laboratory analyses, assuring data quality and allowing for comparisons across waterworks.

In addition, the initial quality control and data pre-processing performed by Voutchkova et al. (2021) assured high data quality and validity for the present study.

### 4.4. Limitations

As a result of the sampling structure, the number of samples collected throughout the period for each waterworks as well as the total number of analyses for each pesticide reported to the national database varied within the study period [14]. The sampling frequency (potentially up to three years between samples) made it difficult to compare waterworks or WSAs over shorter periods of time and made it necessary to evaluate the pesticide status across a 3–4 year period to assure the majority of waterworks or WSAs were presented with a minimum of one sampling event. The gap between samples further introduced a potential uncertainty regarding the pesticide status within the sampling gap.

The high proportion of observations below the limit of detection (<LOD) was addressed by assigning the observations LOD/2 values, while missing observations were substituted by −1. As a result, a high proportion of the input observations for the factor analysis was given the same value and might have influenced the results or their interpretation. Assigning the same value to all observations <LOD might have an influence on the correlation matrix and the resulting factor pattern, whereby the factor structure represents correlations in detected pesticides, but to some extend might also represent the pattern of pesticide analysis, as all pesticides analysed are represented by the same value. From the sensitivity analysis, we found a stable factor pattern indicating that the results were not influenced by the method used to define the pesticide concentration (mean or maximum) or the method used to handle missing and undetected observations.

One of the challenges in this study was the fact that we could only detect what had been analysed in the drinking water. The mandatory monitoring program has changed over time, pesticides have been removed, while others included, in combination with local and national screening programs, meaning the number of pesticides subject for analysis is increasing. Detecting “new” pesticides gives information that the pesticide or metabolite is present in the drinking water at the time of sampling, but we do not know for how long it has been there.

We propose a set of pesticide groups and factors as an alternative to evaluating pesticide exposure by detection or non-detection for all pesticides at once or one pesticide at the time. Hence, our findings may be applicable for exposure assessments in future epidemiological studies of health effects.

The IARC and the United States Environmental Protection Agency (EPA) have evaluated various chemicals including pesticides for carcinogenic effects [11,36,37]. In our dataset, we identified three pesticides classified as potentially carcinogenic to humans (2A and 2B): 2,4-D, glyphosate, and malathion. Both 2,4-D and glyphosate must be analysed given the Danish legislation (Drikkevandsbekendtgørelsen) and have been detected in waterworks in all parts of Denmark, while malathion is not part of the mandatory analyses and was only analysed at 13 waterworks (17 WSAs) over the study period. Furthermore, 2,4-D was detected 9 times and never exceeded the DWQS (maximum concentration of 0.08 µg/L); glyphosate was detected 66 times, of these 16 exceeded DWQS (maximum concentration of 3.20 µg/L); and malathion was detected five times, once exceeding the DWQS (maximum concentration of 0.12 µg/L).

## 5. Conclusions

Over the period 2002–2018, 167 different pesticides and metabolites were analysed and reported to the Danish national database for drinking water quality, 55 of which were found to be above the limit of detection (0.010 µg/L) in some parts of the country. For the study period 2002–2018, we observed decreases in the numbers of analyses and observations above the limit of detection for phenoxy herbicides and benzonitriles and increases for organophosphates. We reduced the data complexity by identifying five factor components in the period 2002–2011 and seven in the period 2012–2018.

The geographic pattern of pesticides identified areas with a high pesticide burden in drinking water and indicated areas with a high vulnerability to pesticides in ground water. Our results extend the previous findings from the study by Voutchkova et al. (2021).

## Figures and Tables

**Table 1:** Resulting factor pattern for the factor analysis for 2002–2011. Loading scores are marked in bold if they meet the criterion of >0.45 or <−0.45.

	Pesticide Group	Factor 1	Factor 2	Factor 3	Factor 4	Factor 5
MCPA	Phenoxy	**0.959**	0.003	−0.001	−0.011	0.001
Dichlorprop	Phenoxy	**0.958**	−0.002	−0.001	0.011	−0.001
Atrazine	Triazine	0.002	**0.817**	0.050	−0.031	−0.011
Atrazine, desethyl-	Triazine	0.001	**0.784**	0.297	−0.012	−0.019
Atrazine, hydroxy-	Triazine	0.002	**−0.632**	**0.458**	−0.073	−0.064
Simazine	Triazine	−0.002	−0.131	**0.820**	0.062	0.066
Atrazine, desisopropy	Triazine	0.000	0.209	**0.733**	−0.013	−0.031
Diuron	Urea	0.014	−0.032	0.024	**0.815**	−0.017
4-CPP	Phenoxy	−0.014	0.037	0.024	**0.807**	0.010
Cyanazine	Triazine	0.001	−0.019	0.073	−0.033	**0.679**
2,4-D	Phenoxy	0.001	0.019	−0.009	−0.004	**0.637**
DNOC	Dinitrophenol	−0.001	0.019	−0.017	0.027	**0.560**
**Variance explained**		2.177	1.841	1.333	1.193	1.097

**Table 2:** Resulting factor pattern for the factor analysis 2012–2018. Loading scores are marked in bold if they meet the criterion of a loading score >0.45 or <−0.45.

	Pesticide Group	Factor 1	Factor 2	Factor 3	Factor 4	Factor 5	Factor 6	Factor 7
2-(2,6-dichlorphenoxy) propanoic acid	Phenoxy acid	**0.988**	−0.010	0.004	0.004	−0.011	−0.011	0.007
Terbuthylazine-desethyl	Triazine	**0.985**	−0.007	0.000	0.019	−0.013	−0.012	0.007
Simazine, hydroxy	Triazine	**0.984**	−0.007	0.000	0.019	−0.013	−0.012	0.007
4-Nitrophenol	Organophosphate	**0.976**	−0.025	0.006	0.015	−0.013	−0.011	0.007
4-CPP	Phenoxy	**0.967**	−0.004	0.004	0.000	−0.008	−0.014	0.005
DEIA	Triazine	**0.959**	−0.014	−0.008	0.022	−0.014	0.072	0.008
AMPA	Organophosphate	**0.749**	−0.007	−0.007	−0.013	0.010	−0.009	−0.042
Glyphosate	Organophosphate	**0.480**	0.010	−0.024	−0.022	0.184	−0.009	−0.091
Metribuzin	Triazinone	0.011	**0.944**	−0.067	−0.078	−0.001	−0.005	0.053
Metribuzin-diketo	Triazinone	−0.044	**0.942**	−0.079	0.124	0.001	−0.004	0.036
Metribuzin-desamino-diketo	Triazinone	−0.044	**0.942**	−0.079	0.124	0.001	−0.004	0.036
Diuron	Phenylurea	0.042	**0.877**	−0.062	−0.073	−0.003	−0.008	0.059
CGA 62826	Acylamino acid	−0.006	**0.742**	0.204	−0.051	0.000	0.019	−0.125
CGA 108906	Acylamino acid	−0.006	**0.742**	0.204	−0.051	0.000	0.020	−0.125
Methyl-desphenyl-chloridazon	Pyridazinone	−0.001	−0.005	**0.916**	0.009	−0.009	0.024	0.043
Desphenyl chloridazon	Pyridazinone	−0.002	−0.014	**0.894**	0.018	−0.008	0.026	0.046
1,2,4-Triazole	Conazole	−0.010	0.065	**0.780**	0.014	0.006	−0.056	−0.028
N,N-dimethylsulfamide (DMS)	Phenylsulfamide	−0.003	0.022	**0.753**	0.012	0.009	−0.052	−0.035
Chloridazon	Pyridazinone	0.004	−0.034	**0.653**	−0.019	0.010	0.050	0.046
Desethyl-hydroxy-atrazine	Triazine	−0.013	−0.014	0.008	**0.992**	0.002	−0.001	0.001
Deisopropyl-hydroxyatrazine	Triazine	−0.013	−0.014	0.008	**0.992**	0.002	−0.001	0.001
2,6-dichlorobenzoic acid	Nitrile herbicides	−0.007	−0.010	0.004	**0.986**	0.002	−0.002	0.000
Didealkyl-hydroxy-atrazine	Triazine	0.284	0.060	0.012	**0.482**	−0.002	0.004	−0.034
Mecoprop	Phenoxy	0.005	0.004	0.006	0.006	**0.971**	0.002	0.020
Dichlorprop	Phenoxy	0.009	−0.014	0.013	0.004	**0.967**	0.002	0.022
MCPA	Phenoxy	0.046	0.009	−0.011	−0.006	**0.960**	−0.001	−0.022
Atrazin, desisopropyl	Triazine	0.000	−0.004	−0.030	0.031	0.001	**0.805**	0.000
Hexazinone	Triazinone	−0.021	0.010	0.024	0.028	0.000	**0.657**	0.000
Atrazine, desethyl-	Triazine	0.027	0.009	−0.002	−0.085	0.001	**0.606**	−0.005
Atrazine	Triazine	−0.014	0.003	0.003	0.018	0.002	**0.504**	0.001
Atrazine, hydroxy-	Triazine	−0.167	−0.097	0.035	0.005	0.014	0.000	**0.902**
Ethylenthiourea	Dithiocarbamate	0.318	0.149	0.036	−0.036	0.003	−0.005	**0.630**
**Variance explained**		7.645	4.540	3.153	2.838	2.705	1.714	1.204

## Data Availability

Data was obtained from the Jupiter database: https://www.geus.dk/produkter-ydelser-og-faciliteter/data-og-kort/national-boringsdatabase-jupiter (accessed on 27 September 2021).

## Supplementary Materials

The following supporting information can be downloaded at: https://www.mdpi.com/article/10.3390/ijerph19020823/s1, Table S1: List of pesticides detected in Danish drinking water in the period 2002–2018, Figure S1. Geographical distribution of Benzothiazinone pesticides, Figure S2. Geographic distribution of Benzonitriles herbicides, Figure S3. Geographical distribution of Organophosphate pesticides, Figure S4. Geographical distribution of Phenoxyacids pesticides, Figure S5. Geographical distribution of Triazine pesticides, Figure S6. Geographical distribution of Triazone pesticides, Figure S7. Areas where multiple pesticide groups have been detected (>DL), Figure S8 (Figure 5f): Geographical distribution of factor components 2002–2011, Figure S9. Scree plot, Table S2. factor pattern, Figure S10. Scree plot, Table S3. factor pattern, Figure S11 scree plot, Table S4. factor pattern, Figure S12. scree plot, Table S5. factor pattern, Figure S13 scree plot, Table S6. factor pattern, Figure S14. scree plot, Table S7. Factor pattern. References [38,39,40,41,42,43,44,45,46,47,48,49] are cited in the Supplementary Materials.
