# Supplementary material for: Geographical Distribution and Pattern of Pesticides in Danish Drinking Water 2002–2018: Reducing Data Complexity
Source: Int J Environ Res Public Health. 2022 Jan 12;19(2):823. doi: 10.3390/ijerph19020823 (PMC8775924; doi:10.3390/ijerph19020823)
Supplement: Supplementary file 1 [file ijerph-19-00823-s001.zip › ijerph-1419224-supplementary.pdf]

Supplementary material: Geographical distribution and pattern of pesticides in Danish drinking water 2002-2018: reducing data complexity

**Table S1.** List of pesticides detected in Danish drinking water in the period 2002-2018.Type	Pesticide/ Metabolite (*)	Pesticide Name	Analysed	WW/WSA	No. Measurements >LOD/>LOQ/>DWQS	Approval Status (Pesticide) in DK	Characteristics
Hrb, phenoxypropionic	4-CPP *	Dichlorprop, mecoprop	2002–2018	2588/2318	63/24/8		
Hrb, triazine	DEIA *	Atrazine, terbuthylazine, simazine (other chlorotriazines)	2004, 2007–2018	2531/2307	156/42/7		Metabolite of multiple pesticides
Hrb, triazine	Terbuthylazine-desethyl *	Terbuthylazine	2002–2018	2543/2309	2/0/0		
Hrb, triazine	Simazine, hydroxy *	Simazine	2002–2018	2532/2307	49/29/2		
Hrb, phenylurea	Metoxuron	Metoxuron	2002–2011	10/14	1/0/0		
Hrb, nitrile herbicides	Dichlobenil	Dichlobenil, chlorthiamid	2002–2018	2890/2424	8/0/0	Not approved after 1996 [38]	Applied on fruit trees
Hrb, phenylurea	Diuron	Diuron	2002–2018	2729/2364	16/0/0	Not approved after 2005 [39]	Used in garden nursery and for cut greenery
Hrb, nitrile herbicides	2,6-Dichlorobenzamide (BAM) *	Dichlobenil, chlorthiamid	2002–2018	3019/2460	5605/2422/327		Applied in orchards
Ins, organophosphate	4-Nitrophenol *	Parathion	2002, 2004, 2007–2018	2530/2307	56/19/1	Banned for use in EU from 2003 [40]	
phenoxyacid	2-(2,6-dichlorophenoxy) propanoic acid [2,6-DCPP] *	(Phenoxyacid)	2002–2018	2551/2311	40/4/0		
Ins, organophosphate	Malathion	Malathion	2002–2011, 2017–2018	13/17	5/4/1	Banned for use in EU after 2007, later re-approved for restricted use in EU from 2011 [41,42]	
Hrb, phenoxypropionic	TFMP *	Fluazifop-P-butyl	2012–2018	13/17	1/0/0		
Hrb, triazine	Atrazine, desethyl- *	Atrazine	2002–2018	3013/2460	288/73/7		
Hrb, triazine	Atrazine, desisopropyl *	Atrazine	2002–2018	3012/2460	229/32/4		

Hrb, triazine	Atrazine, hydroxy- *	Atrazine	2002–2018	3010/2460	48/5/1		
Hrb, pyridazinone	Chloridazon	Chloridazon	2002–2012, 2016–2018	1174/1098	2/0/0	Restricted 2012 [43]	
Hrb, pyridine herbicide	Clopyralid	Clopyralid	2002–2007, 2011, 2018	22/57	6/5/0	Restricted after 2012 [43]	
Hrb, triazine	Cyanazine	Cyanazine	2002–2012, 2014–2018	2938/2430	3/0/0	Not approved after 1994 [44]	
Ins, organophosphate	Dimethoate	Dimethoate, Formothion	2002–2012, 2014, 2016–2018	2936/2429	3/0/0	Approval removed in EU after 2011 [45], banned in DK after 2012 [43]	
Fun, dithiocarbamate	Ethylenthiourea *	Maneb, zineb, mancozeb	2002, 2004, 2010–2018	2519/2303	7/1/0		
Hrb, organophosphate	Glyphosate	Glyphosate	2002–2018	2536/2308	66/37/16		
Hrb, triazinone	Hexazinone	Hexazinone	2002–2018	3013/2460	206/52/2	Not approved after 1994 [44]	
Hrb, triazinone	Metamitron	Metamitron	2002–2018	2937/2429	7/2/0	Not approved after 2016	
Hrb, triazinone	Metribuzin	Metribuzin	2002–2018	2446/2242	2/0/0	Not approved after 2005 [46]	Used in potato production
Hrb, dinitroaniline	Pendimethalin	Pendimethalin	2002–2018	2938/2430	16/3/0	Restricted 2012 [43]	
Fun, conazole (imidazol)	Prochloraz	Prochloraz	2002–2011, 2017–2018	12/16	1/0/0	Not approved after 2011 [40]	
Hrb, triazine	Terbuthylazine	Terbuthylazine	2002–2018	2830/2345	13/2/0	Regulated in 2003, Not approved after 2009 [40]	
Fun, conazole (triazoles)	1,2,4-Triazole *	Epoxiconazole, Difenoconazole, Propiconazole, Tebuconazole	2015, 2018	1155/1122	7/0/0		
Hrb, triazinone	Metribuzin-desamino-diketo *	Metribuzin	2004, 2006–2018	2431/2233	80/3/0		Used in potato production
Hrb, triazinone	Metribuzin-diketo *	Metribuzin	2004, 2006–2018	2431/2233	1/0/0		Used in potato production
Hrb, triazine	Desethyl-hydroxy-atrazine *	Atrazine	2004, 2010–2018	2498/2286	6/0/0		
Hrb, triazine	Deisopropyl-hydroxyatrazine *	Atrazine	2004, 2010–2018	2498/2286	8/0/0		

Hrb, triazine	Didealkyl-hydroxy-atrazine *	Atrazine	2004, 2010–2018	2515/2299	38/15/0		
Hrb, triazine	Terbutylazine,hydroxy *	Terbuthylazine	2002–2018	283/251	3/0/0		
Hrb, nitrile herbicides	2,6-dichlorobenzoic acid *	Dichlobenil, chlorthiamid (BAM)	2002–2018	2505/2286	53/21/0		Applied on fruit trees
Hrb, phenoxypropionic	Dichlorprop	Dichlorprop	2002–2018	3012/2460	198/82/9	Not approved after 1996 [38]	
Hrb, phenoxyacid	MCPA	MCPA, MCPB, MCPA-thioethyl	2002–2018	3012/2460	36/12/4	Not approved after 1996 [38]	
Hrb, phenoxypropioinic	Mecoprop	Mecoprop	2002–2018	3013/2460	142/52/7	Not approved after 1996 [38]	
Hrb, Ins, Fun; dinitrophenol	DNOC	DNOC	2002–2018	2938/2430	5/1/0	Not approved after 2002 [47]	
Hrb, dinitrophenol	Dinoseb	Dinoseb	2002–2018	2938/2430	8/1/0	Not approved after 1992 [48]	
Hrb, triazine	Atrazine	Atrazine	2002–2018	3012/2460	163/43/4	Not approved after 1994 [44]	
Hrb, triazine	Simazine	Simazine	2002–2018	3012/2460	75/16/0	Not approved after 2005 [40]	
Hrb, organophosphate	AMPA *	Glyphosate	2002–2018	2547/2309	20/8/3		
Hrb, pyridazinone	Desphenyl chloridazon *	Chloridazon	2010, 2016–2018	1729/1614	729/485/163		
Hrb, pyridazinone	Methyl-desphenyl-chloridazon *	Chloridazon	2010, 2017–2018	1671/1564	96/29/6		
Fun, anilide fungicide	CGA 108906 * [N-(2-carboxy-6-methylphenyl) -N-methoxyacetyl)-DL-alanine]	Metalaxyl/Metalaxyl-M	2010, 2014–2017	2242/2086	98/35/6	Not approved after 2003 [47]	Used in potato production
Fun, anilide fungicide	CGA 62826 * [N-(2,6-dimethylphenyl)- N-(2-methoxyacetyl)-DL-alanine]	Metalaxyl/Metalaxyl-M	2010, 2014–2018	2242/2086	64/54/0		Used in potato production
Fun, phenylsulfamide	N,N-dimethylsulfamide [DMS] *	Tolylfluanid, dichlofluanid	2010, 2018	1029/1002	364/222/36		Production of fruit, berries, and tomatoes
Hrb, chloroacetanilide	Metazachlor ESA *	Metazachlor	2018	27/68	5/4/0		
Hrb, chloroacetanilide	Metazachlor OA *	Metazachlor	2018	27/68	5/4/0		

Hrb, chloroacetanilide	Dimethachlor ESA *	Dimethachlor	2018	27/68	6/4/0		Applied on rapeseed
Hrb, chloroacetanilide	Propachlor ESA *	Propachlor	2018	27/68	1/0/0	Not approved after 1994 [44]	
Hrb, phenoxyacid	2,4-D	2,4-D; 2,4-DB; 2,4-D-dimethylammonium; 2,4-DEP	2002–2018	2988/2453	9/2/0	Not approved after 1996 [38]	
Hrb, benzothiazinone	Bentazone	Bentazone	2002–2018	3012/2460	605/214/37	Restricted 2012 [43]	
Hrb, phenylurea	Isoproturon	Isoproturon	2002–2018	2937/2430	14/4/1	Not approved after 1999 [49]	

Pesticide type is defined by the parent pesticide (pesticide name). Metobolites are indicated with an "*". Information on period when the pesticides have been analysed, number of waterworks and water supply areas represented, and number of analyses where the pesticide have exceeded the LOD, LOQ and DWQS. Further approval status in DK and EU indicate the period of use.

1. Geographical distribution of pesticides groups

1.1. Benzothiazinone

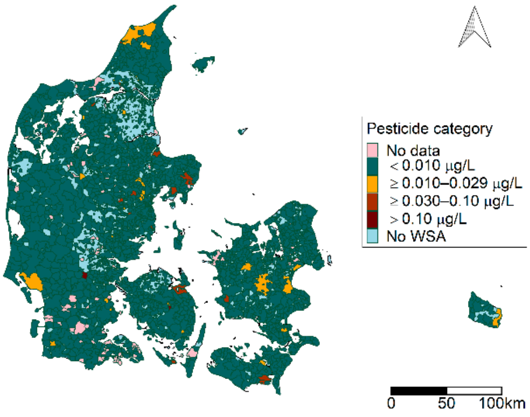

(a)

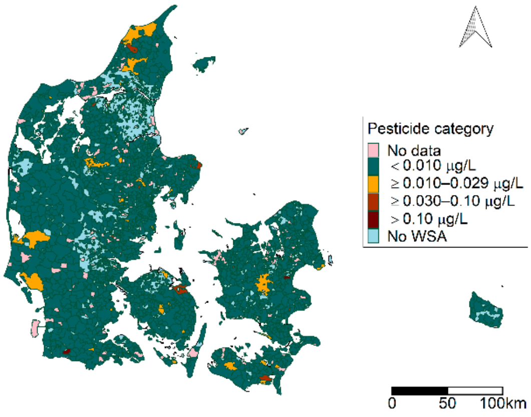

(b)

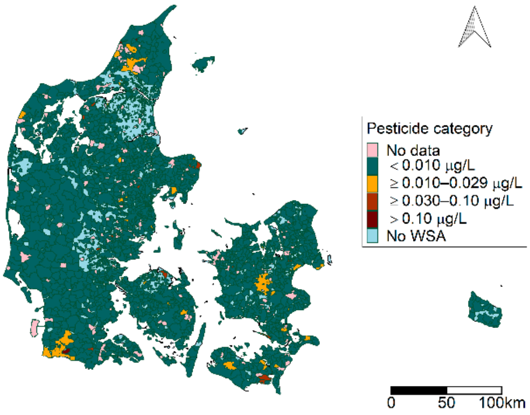

(c)

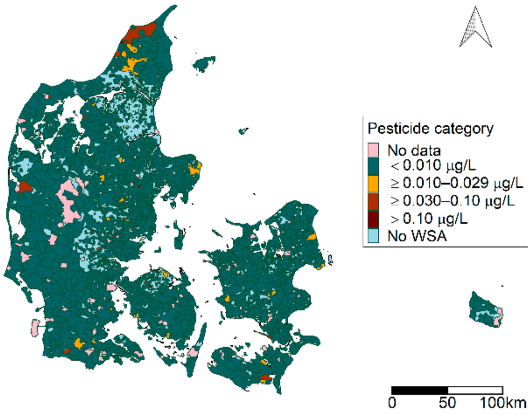

(d)

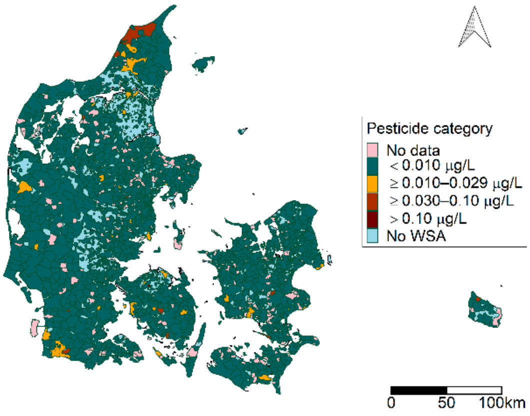

(e)

Figure S1. Geographical distribution of Bentazoan pesticides, a) 2002-2005, b) 2006-2008, c) 2009-2011, d) 2012-2015, e) 2016-2018.

1.2. Benzonitriles herbicides

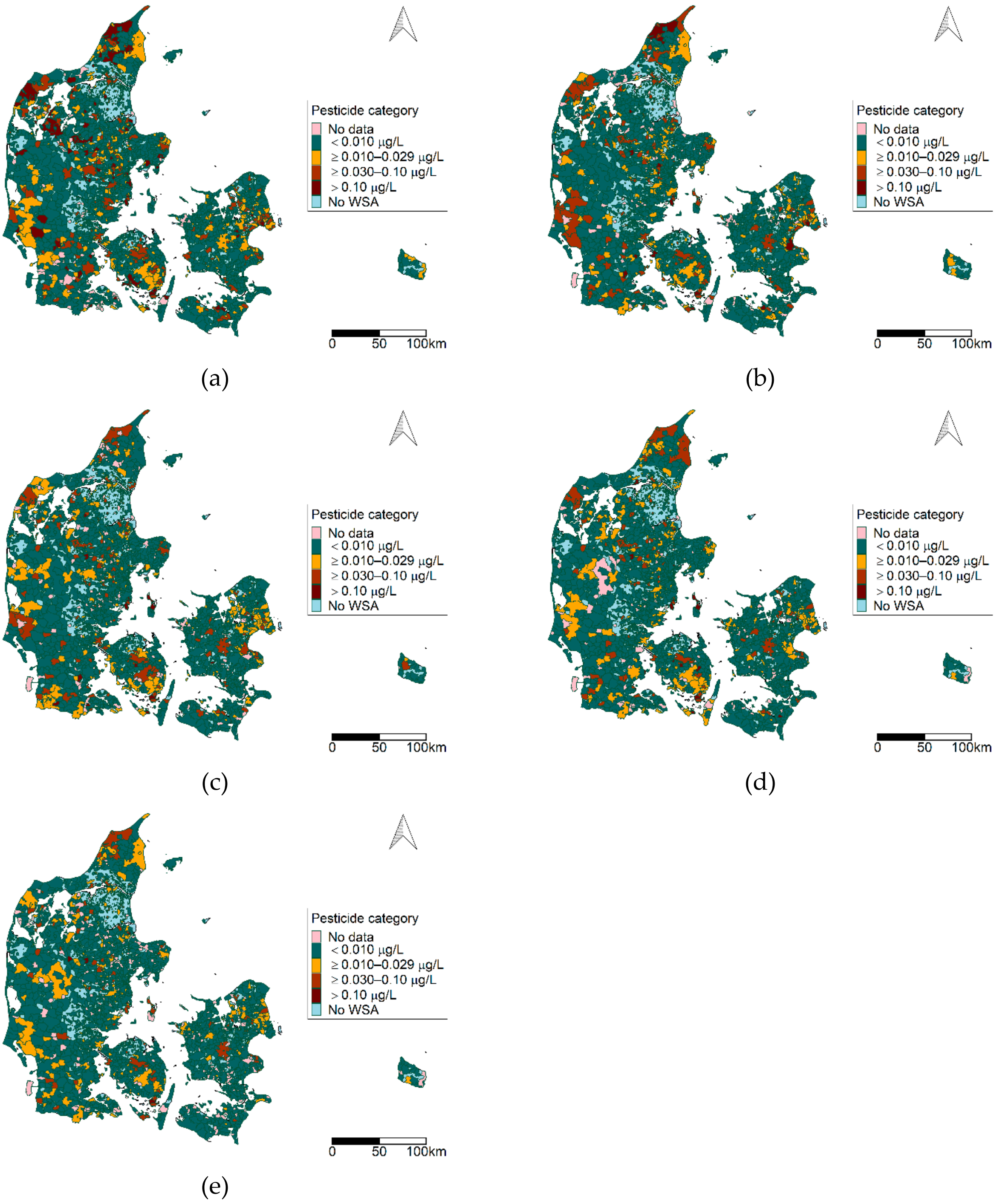

Figure S2. Geographic distribution of Nitril pesticides, a) 2002-2005, b) 2006-2008, c) 2009-2011, d) 2012-2015, e) 2016-2018.

1.3. Organophospates

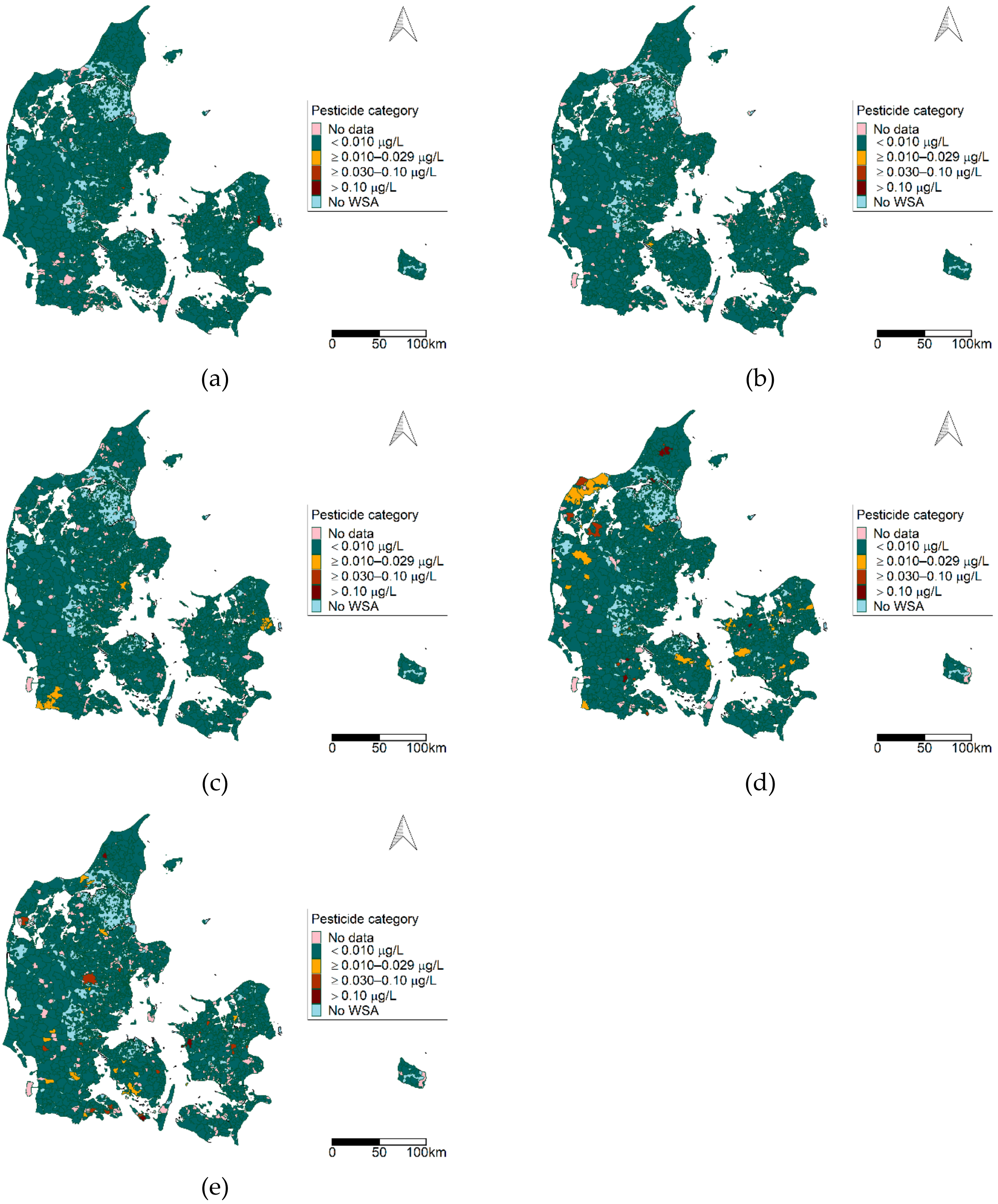

Figure S3. Geographical distribution of Organophosphate pesticides, a) 2002-2005, b) 2006-2008, c) 2009-2011, d) 2012-2015, e) 2016-2018.

1.4. Phenoxyacids herbicides

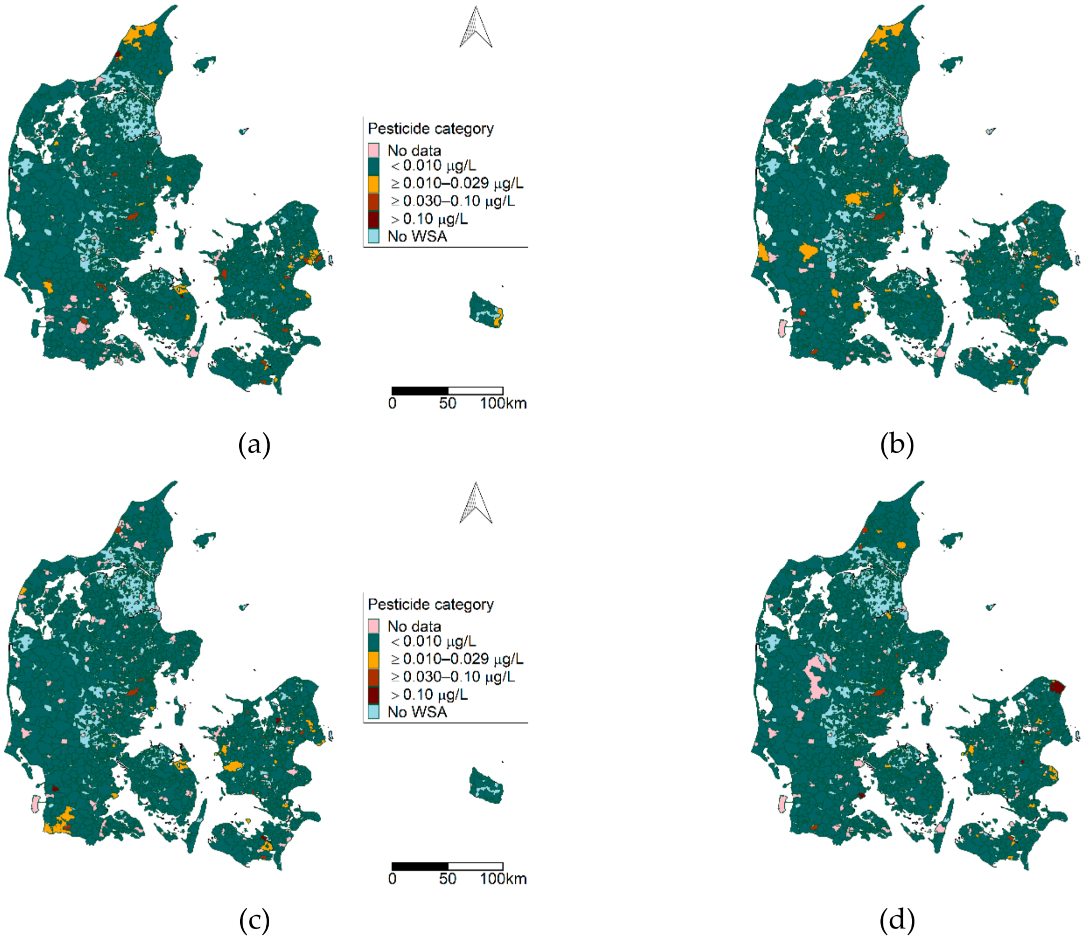
Pesticide category
No data
< 0.010 µg/L
≥ 0.010–0.029 µg/L
> 0.030–0.10 µg/L
> 0.10 µg/L
No WSA
0
50
100km
(a)
(b)
(c)
(d)

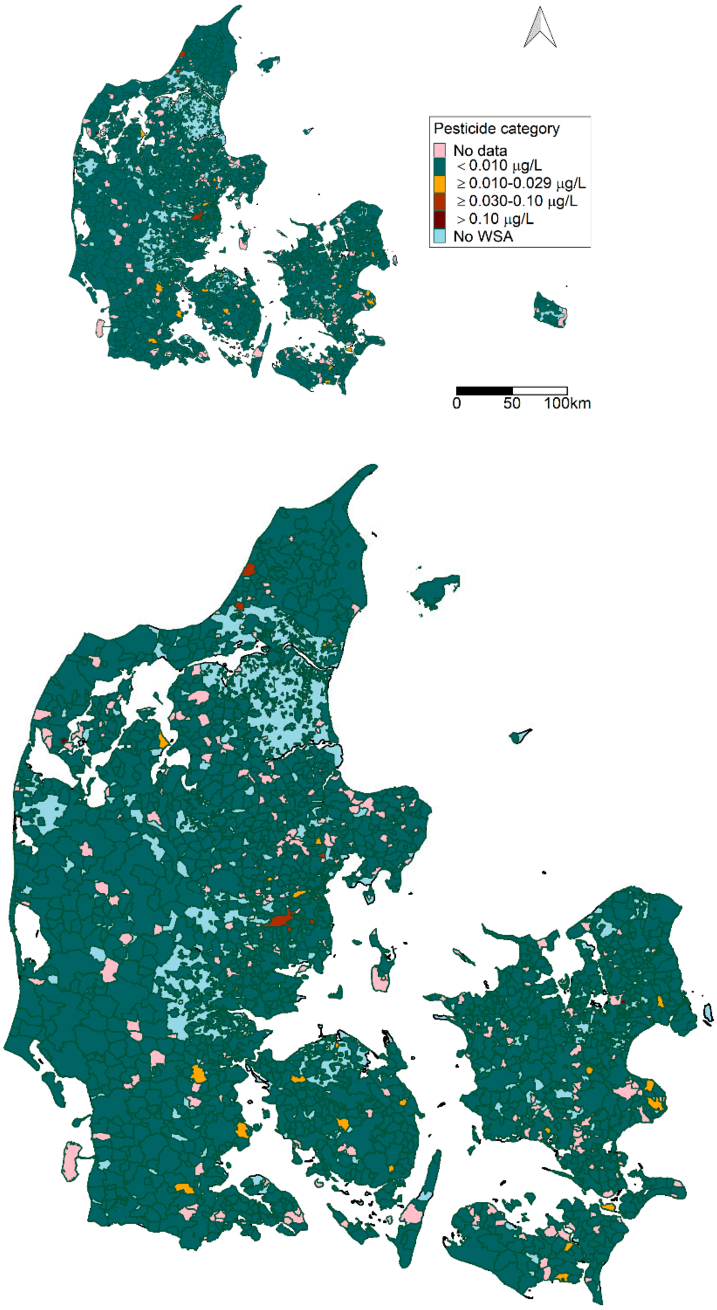

(e)

Figure S4. Geographical distribution of Phenoxyacids pesticides, a) 2002-2005, b) 2006-2008, c) 2009-2011, d) 2012-2015, e) 2016-2018.

1.5. Triazine herbicides

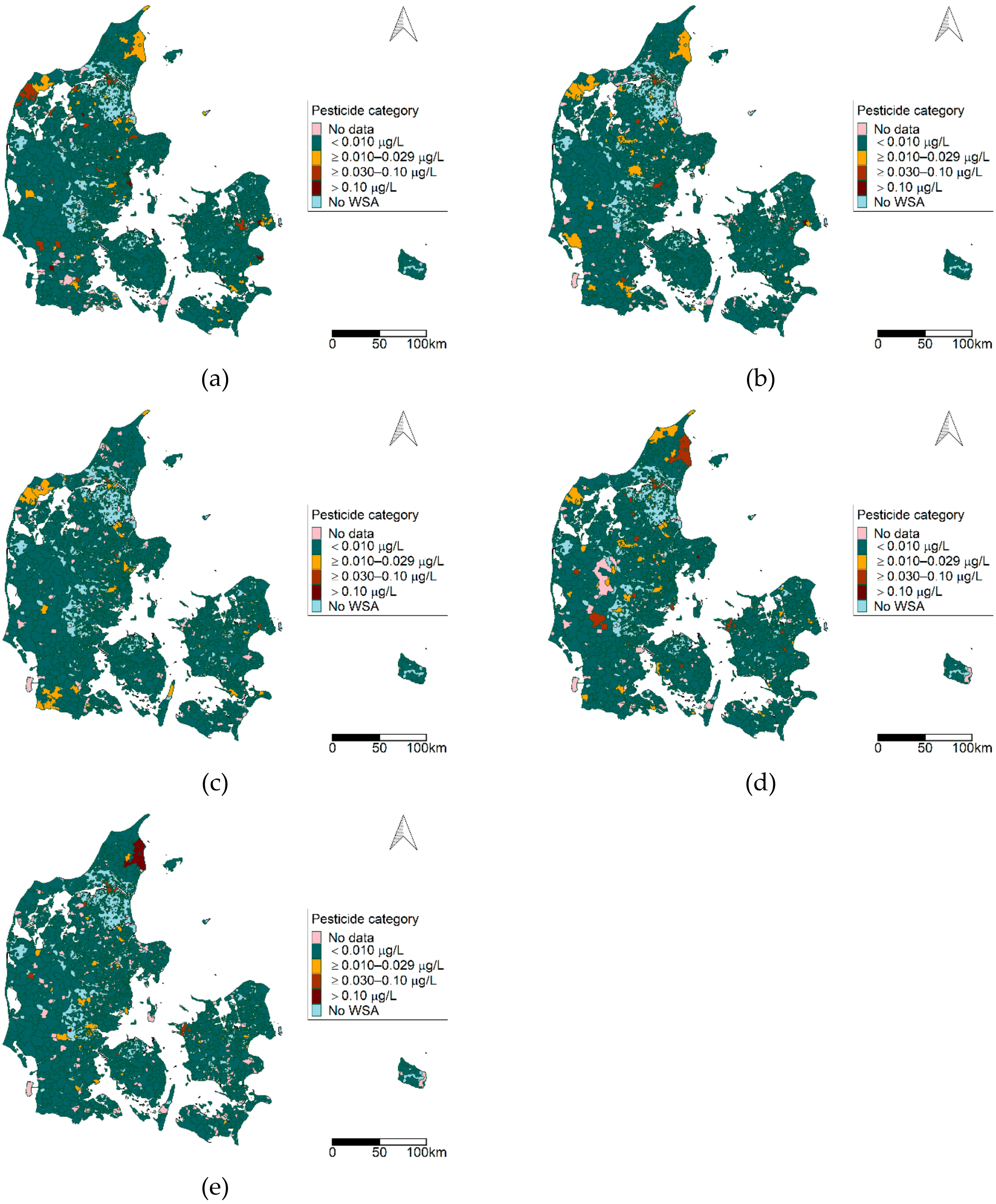

Figure S5. Geographical distribution of Triazine pesticides, a) 2002-2005, b) 2006-2008, c) 2009-2011, d) 2012-2015, e) 2016-2018.

1.6. Triazinone herbicides

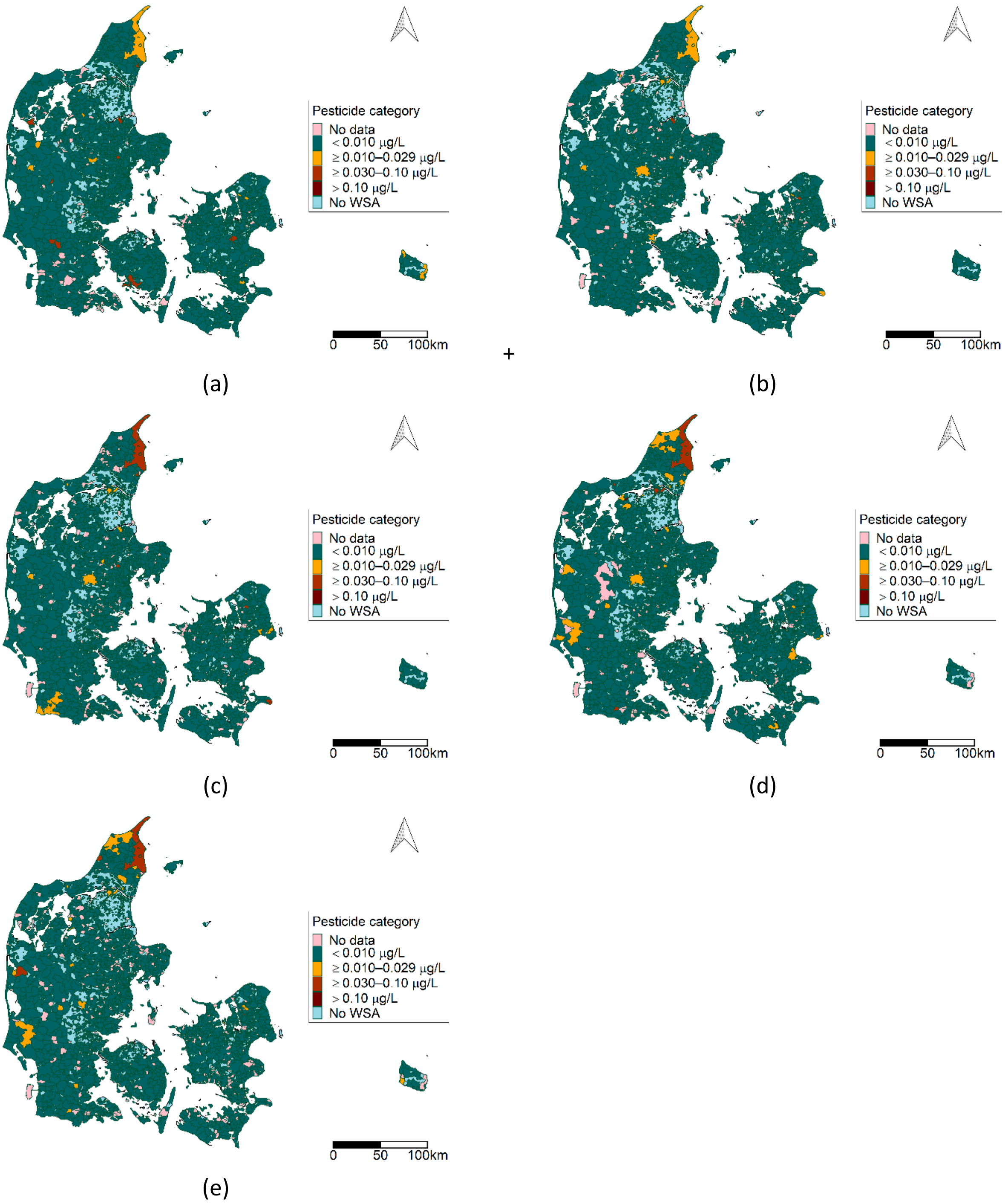

Figure S6. Geographical distribution of Triazone pesticides, a) 2002-2005, b) 2006-2008, c) 2009-2011, d) 2012-2015, e) 2016-2018.

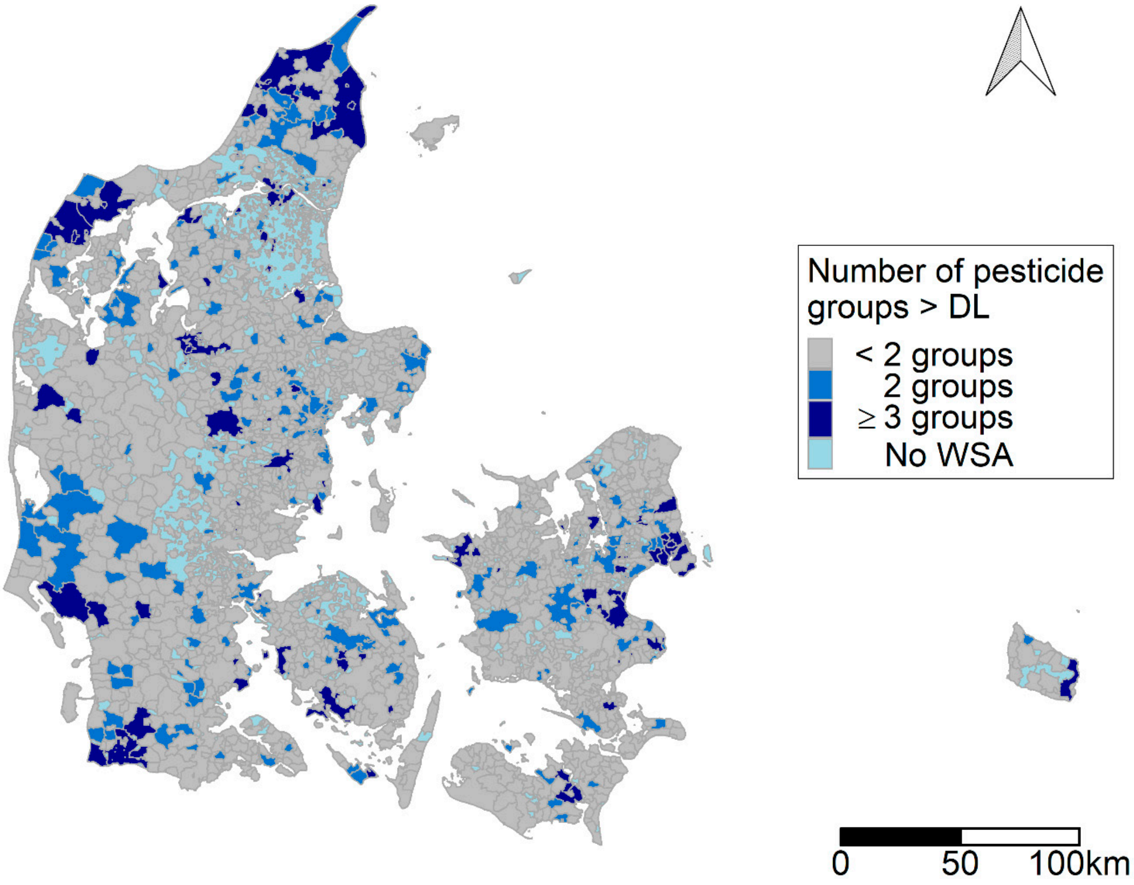

Number of pesticide groups > DL
< 2 groups
2 groups
≥ 3 groups
No WSA
0
50
100km

(a)

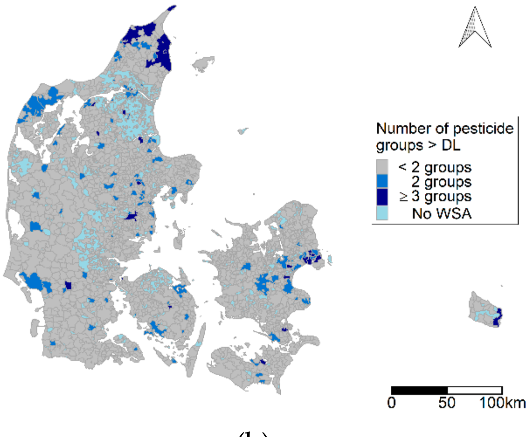

Number of pesticide groups > DL
< 2 groups
2 groups
≥ 3 groups
No WSA
0
50
100km

(b)

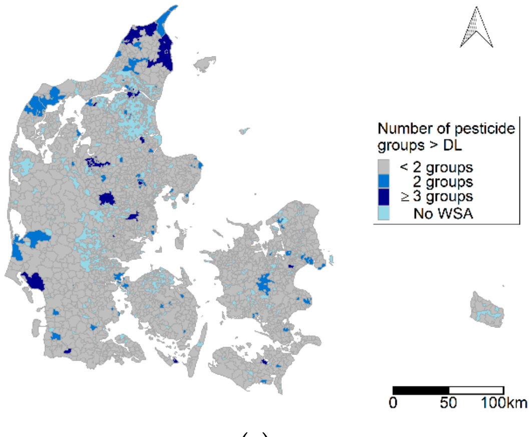

Number of pesticide groups > DL
< 2 groups
2 groups
≥ 3 groups
No WSA
0
50
100km

(c)

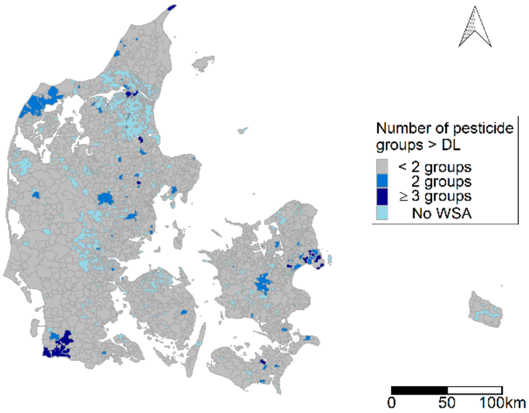

(d)

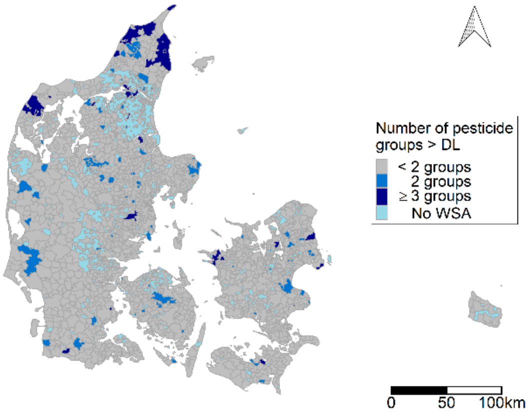

(e)

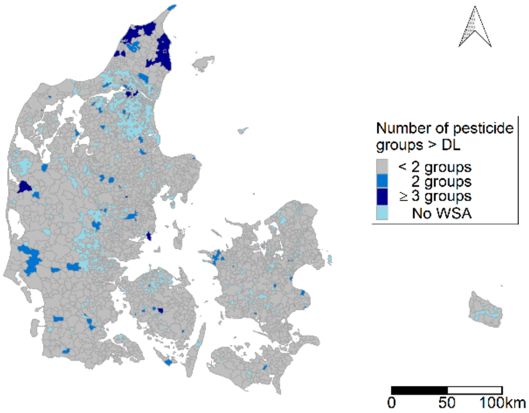

(f)

Figure S7. Areas where multiple pesticide groups have been detected (>DL). a) 2002-2018, b) 2002-2005, c) 2006-2008, d) 2009-2011, e) 2012-2015, f) 2016-2018.

2. Factor analysis – 2002-2011

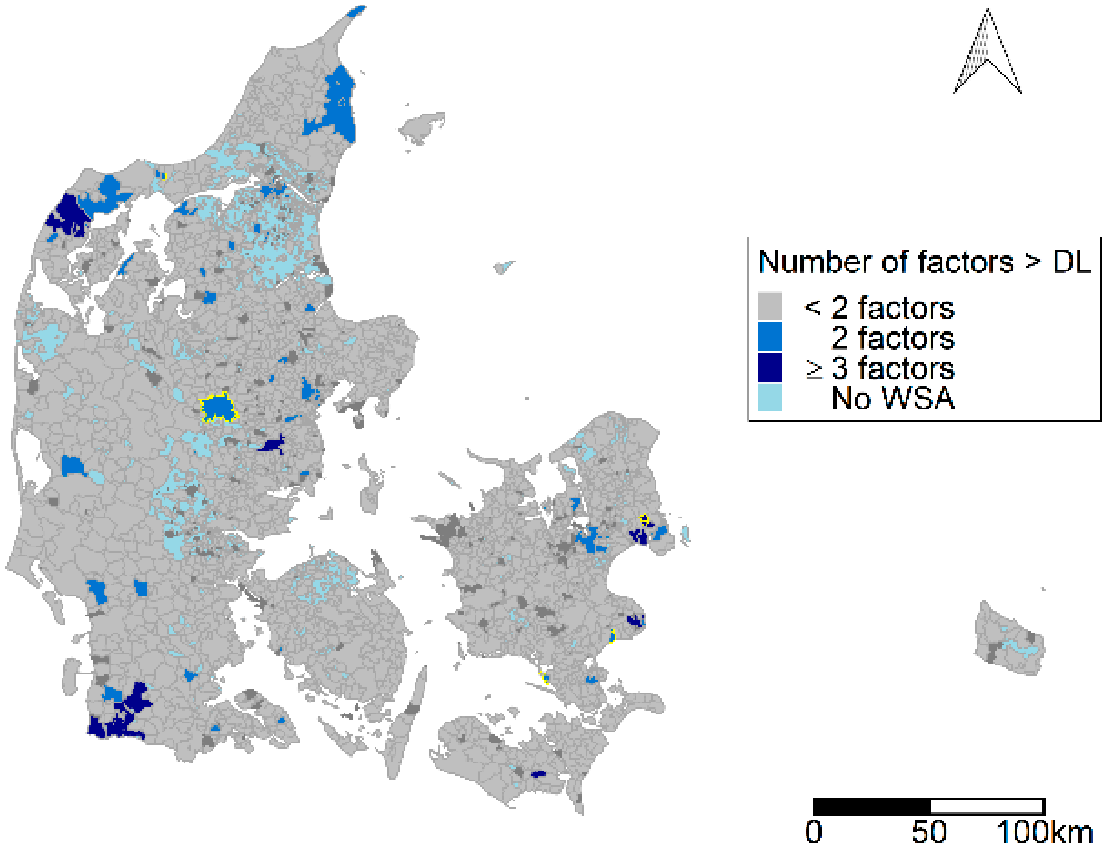

Figure S8 (Figure 5f). WSA where multiple factor components have been identified. WSAs where the pesticide hydroxyatrazine define the factor status is indicated by a yellow border. Hydroxyatrazine represents both factor 2 and 3.

3. Factor analysis – sensitivity analysis

To analyse and account for the effect of the choices made regarding data structure on the final factor pattern a set of sensitivity analyses have been made with an alternative substitution of missing observations and pesticide concentration/category.

Missing observations: in the main analysis, missing observations were substituted with -1, as a sensitivity analysis, missing observations were substituted with 0.

Pesticide concentration: the main analysis was made with the maximum concentration measured at the waterworks, as a sensitivity analysis the concentration was defined as the mean concentration measured, further the pesticide concentration was categorised by measurements > QL, and further > DWQS, as 0/1 variable.

3.1. 2002-2011

3.1.1. µg mean, missing =-1

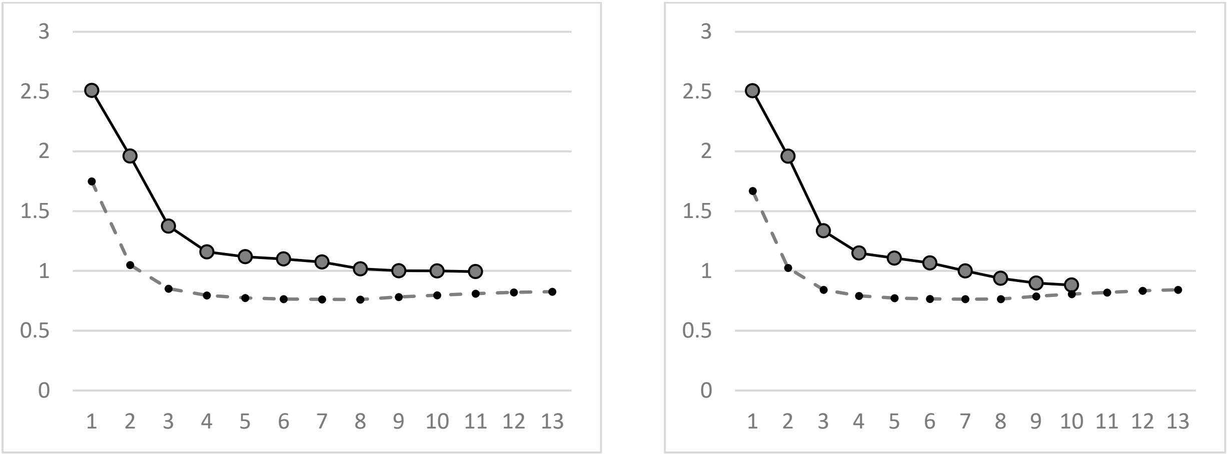

(a) (b)

Figure S9. a) initial scree plot, where 6 factor components were retained, b) final scree plot, where 6 factor components were retained.

The analysis was completed by two iterations.

Table S2. factor pattern

		Factor1	Factor2	Factor3	Factor4	Factor5	Factor6
Atrazine, desethyl-	Triazine	**0.865**	0.009	0.134	0.007	-0.037	-0.012
Atrazine	Triazine	**0.756**	-0.001	0.139	-0.021	0.012	-0.011
2,6-Dichlorobenzamide	Nitrile herbicides	**0.631**	-0.046	-0.030	-0.036	0.251	0.051
Atrazine, hydroxy-	Triazine	**-0.621**	-0.043	0.429	-0.066	0.196	0.029
Dichlorprop	Phenoxy	-0.002	**0.964**	0.003	0.005	0.106	0.001

MCPA	Phenoxy	-0.005	**0.955**	0.013	-0.014	-0.065	-0.003
Simazine	Triazine	-0.068	0.018	**0.840**	0.038	-0.075	-0.006
Atrazine, desisopropy	Triazine	0.318	0.002	**0.681**	-0.003	-0.010	-0.006
Diuron	Urea	-0.025	0.009	-0.002	**0.814**	0.044	0.005
4-CPP	Phenoxy	0.013	-0.018	0.037	**0.808**	-0.011	0.025
Bentazone	Benzothiazinone	0.065	-0.113	-0.054	0.016	**0.801**	-0.031
Mecoprop	Phenoxy	0.013	0.244	-0.029	0.023	**0.586**	0.003
Dichlobenil	Nitrile herbicides	0.040	0.009	-0.009	-0.074	0.001	**0.739**
Dimethoat	Organophosphate	-0.017	-0.004	0.000	0.055	-0.028	**0.528**
Isoproturon	Phenylurea	-0.016	-0.008	-0.002	0.056	-0.008	**0.526**

3.1.2. μg max, missing = 0

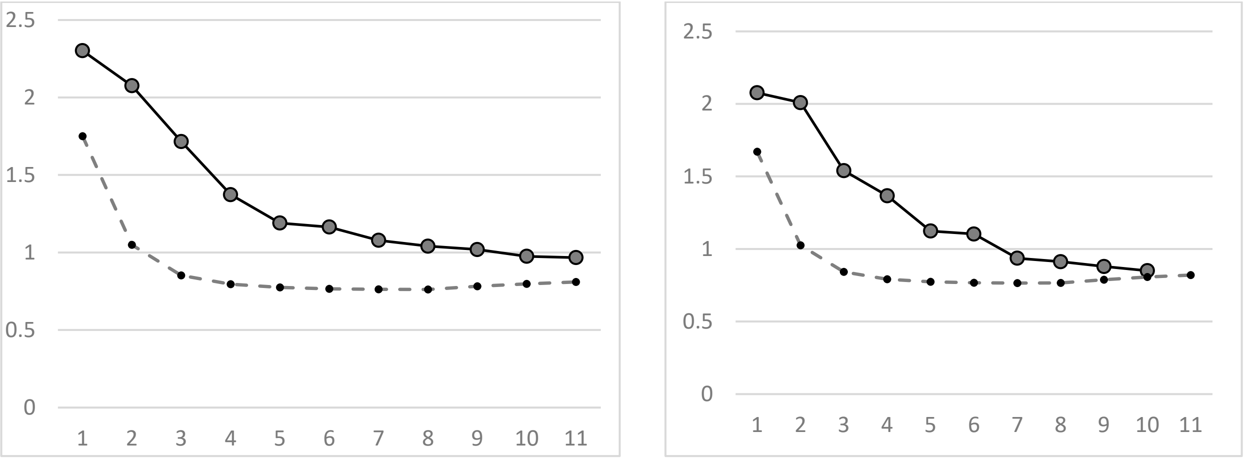

(a) (b)

Figure S10. a) initial scree plot, where 7 factor components were retained, b) final scree plot, where 6 factor components were retained.

The analysis was completed by four iterations.

Table S3. factor pattern

		**Factor1**	**Factor2**	**Factor3**	**Factor4**	**Factor5**	**Factor6**
Atrazine, desethyl-	Triazine	**0.923**	0.001	-0.003	-0.015	0.020	-0.004
Atrazine	Triazine	**0.812**	-0.002	0.011	-0.046	-0.007	0.009
Atrazine, desisopropy	Triazine	**0.679**	0.004	-0.007	0.046	-0.014	-0.009
MCPA	Phenoxy	0.004	**0.990**	-0.156	-0.005	0.011	-0.016
Dichlorprop	Phenoxy	-0.002	**0.920**	0.189	-0.002	-0.004	0.010
4-CPP	Phenoxy	0.013	-0.068	**0.882**	-0.016	0.066	-0.048
Mecoprop	Phenoxypropioinic	-0.013	0.067	**0.860**	0.036	-0.060	0.042
Dimethoat	Organophosphate	0.043	-0.003	0.026	**0.764**	0.027	0.045

Cyanazine	Triazine	0.003	-0.007	0.013	**0.661**	-0.143	-0.020
DNOC	Dinitrophenol	-0.030	-0.003	-0.004	**0.470**	0.061	-0.044
Dinoseb	Dinitrophenol	-0.037	0.003	-0.008	**0.462**	0.101	-0.001
Diuron	Urea	-0.018	0.000	0.055	-0.121	**0.791**	-0.001
Terbuthylazine	Triazine	0.020	0.010	-0.052	0.196	**0.699**	0.008
Bentazone	Benzothiazinone	0.004	-0.010	0.031	-0.092	-0.018	**0.767**
Isoproturon	Phenylurea	-0.008	0.002	-0.041	0.077	0.026	**0.723**

3.1.3.μg mean, missing = 0

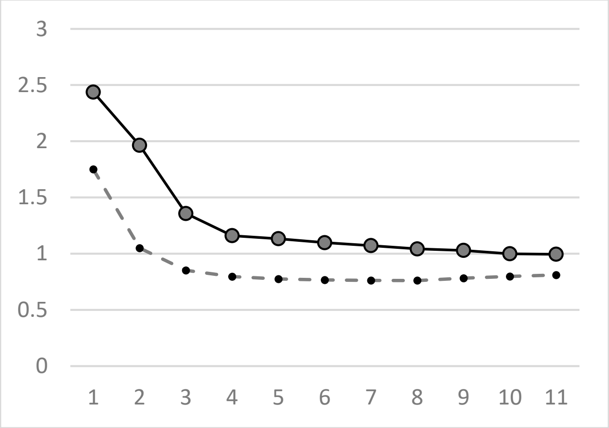

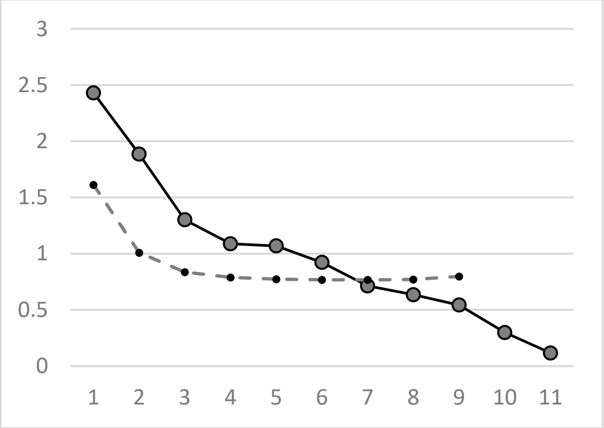

(a) (b)

Figure S11. a) initial scree plot, where 6 factor components were retained, b) final scree plot, where 5 factor components were retained.

The analysis was completed by five iterations.

Table S4. factor pattern

		Factor1	Factor2	Factor3	Factor4	Factor5
Atrazine, desethyl-	Triazine	**0.808**	-0.001	0.189	0.018	-0.013
2,6-Dichlorobenzamide	Nitrile herbicides	**0.802**	0.000	-0.243	-0.012	0.037
Atrazine	Triazine	**0.793**	-0.001	0.091	0.015	-0.020
MCPA	Phenoxy	0.000	**0.971**	0.004	-0.018	-0.005
Dichlorprop	Phenoxy	-0.001	**0.970**	-0.001	0.019	0.005
Simazine	Triazine	-0.143	0.003	**0.911**	-0.002	0.016
Atrazine, desisopropy	Triazine	0.324	0.000	**0.659**	-0.025	-0.004
Diuron	Urea	0.009	0.005	-0.049	**0.808**	-0.017
4-CPP	Phenoxy	0.007	-0.004	0.029	**0.803**	0.019
Dichlobenil	Nitrile herbicides	0.088	0.010	-0.084	-0.054	**0.736**
Dimethoat	Organophosphate	-0.081	-0.010	0.103	0.056	**0.730**

3.2. 2012-2018

3.2.1.µg mean, missing = -1

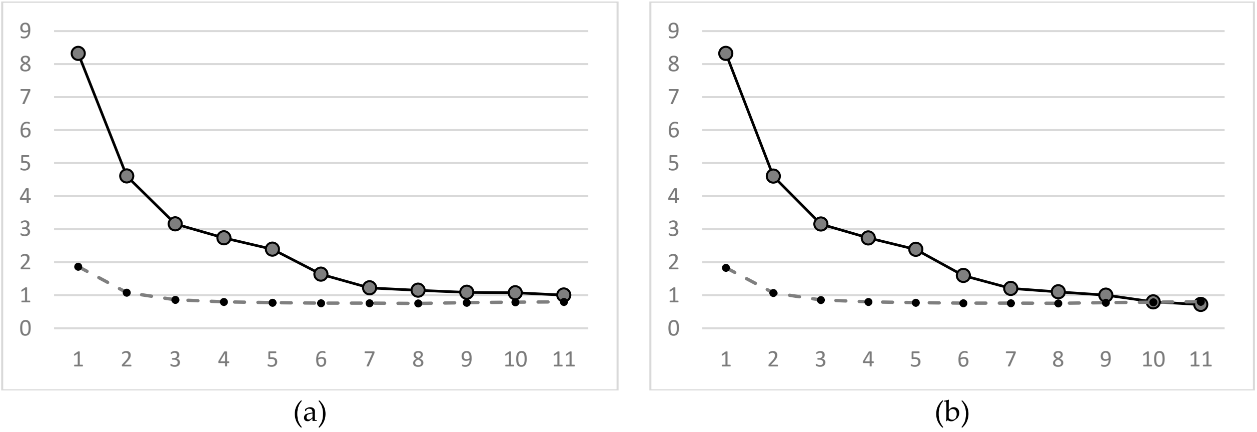

Figure S12. a) initial scree plot, where 8 factor components were retained, b) final scree plot, where 7 factor components were retained.

The analysis was completed by three iterations.

Table S5. factor pattern

		Factor1	Factor2	Factor3	Factor4	Factor5	Factor6	Factor7
2-(2,6-dichlorphenoxy) propanoic acid	Phenoxy acid	**0.987**	-0.007	0.002	0.010	-0.017	-0.008	0.004
4-Nitrophenol	Organophosphate	**0.986**	-0.010	0.001	0.014	-0.017	-0.007	0.004
Terbuthylazine-desethyl	Triazine	**0.985**	-0.006	0.001	0.017	-0.017	-0.007	0.004
Simazine, hydroxy	Triazine	**0.985**	-0.006	0.001	0.017	-0.017	-0.008	0.004
4-CPP	Phenoxy	**0.980**	-0.004	0.003	0.001	0.000	-0.008	0.003
DEIA	Triazine	**0.973**	-0.012	-0.005	0.020	-0.017	0.062	0.005
AMPA	Organophosphate	**0.890**	-0.015	-0.001	-0.012	0.003	-0.012	-0.030
Glyphosate	Organophosphate	**0.830**	-0.011	-0.011	-0.015	0.107	-0.014	-0.040
Metribuzin	Triazinone	0.013	**0.945**	-0.066	-0.078	-0.001	0.010	0.049
Metribuzin-diketo	Triazinone	-0.043	**0.942**	-0.079	0.124	0.002	0.008	0.033
Metribuzin-desamino-diketo	Triazinone	-0.043	**0.942**	-0.078	0.124	0.002	0.008	0.033
Diuron	Phenylurea	0,043	**0.877**	-0.061	-0.073	-0.003	0.008	0.055
CGA 62826	Acylamino acid	-0,011	**0.742**	0.202	-0.052	0.001	-0.018	-0.117
CGA 108906	Acylamino acid	-0,011	**0.742**	0.202	-0.052	0.001	-0.018	-0.117
Methyl-desphenyl-chloridazon	Pyridazinone	-0,001	-0.004	**0.917**	0.010	-0.010	0.022	0.044
Desphenyl chloridazon	Pyridazinone	-0,003	-0.014	**0.897**	0.019	-0.010	0.020	0.049
1,2,4-Triazole	Conazole	-0.007	0.065	**0.780**	0.014	0.005	-0.023	-0.033

N,N-dimethylsulfamide (DMS)	Phenylsulfamide	0.000	0.023	**0.752**	0.013	0.009	-0.019	-0.041
Chloridazon	Pyridazinone	0.009	-0.033	**0.653**	-0.021	0.009	0.007	0.048
Desethyl-hydroxy-atrazine	Triazine	-0.010	-0.015	0.008	**0.991**	0.001	-0.002	0.002
Deisopropyl-hydroxyatrazine	Triazine	-0.010	-0.015	0.008	**0.991**	0.001	-0.002	0.002
2,6-dichlorobenzoic acid	Nitrile herbicides	-0.005	-0.011	0.004	**0.986**	0.001	-0.003	0.002
Didealkyl-hydroxy-atrazine	Triazine	0.280	0.060	0.012	**0.484**	-0.001	-0.003	-0.032
Dichlorprop	Phenoxy	-0.002	-0.006	0.025	-0.010	**0.929**	0.003	0.011
Mecoprop	Phenoxy	-0.007	0.005	-0.007	0.013	**0.885**	0.002	0.010
MCPA	Phenoxy	0.031	0.003	-0.015	0.000	**0.855**	-0.002	-0.014
Atrazine, desisopropy	Triazine	0.001	-0.009	-0.011	0.020	0.001	**0.794**	-0.002
Atrazine	Triazine	-0.013	0.004	0.027	0.011	0.003	**0.732**	-0.012
Atrazine, desethyl-	Triazine	0.008	0.007	-0.008	-0.042	0.000	**0.652**	-0.002
Atrazine, hydroxy-	Triazine	-0.143	-0.099	0.033	0.007	0.009	-0.009	**0.903**
Ethylenthiourea	Dithiocarbamate	0.330	0.149	0.035	-0.033	-0.004	-0.004	**0.630**

3.2.2. μg max, missing = 0

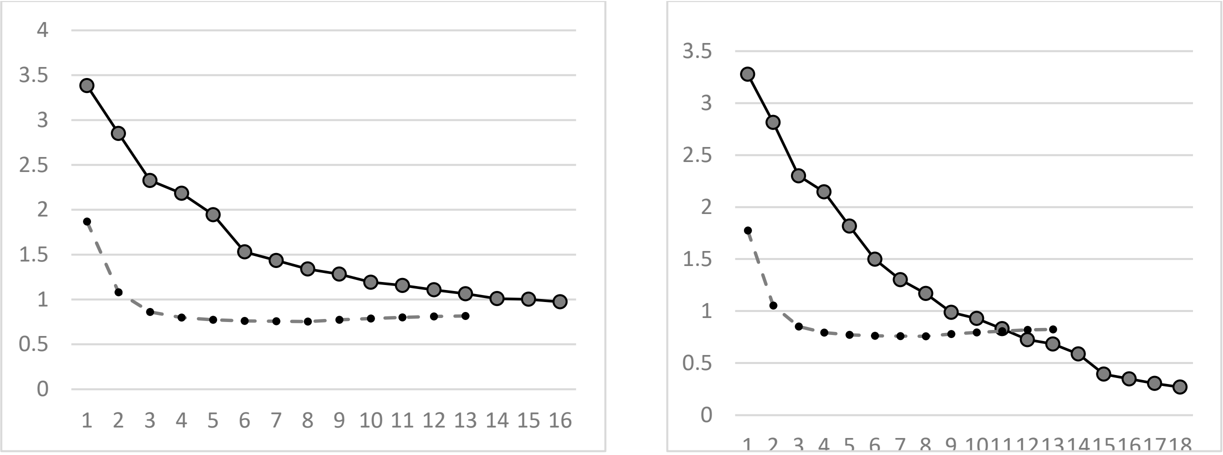

(a) (b)

Figure S13. a) initial scree plot, where 10 factor components were retained, b) final scree plot, where 8 factor components were retained.

The analysis was completed by four iterations.

Table S6. factor pattern

		Factor1	Factor2	Factor3	Factor4	Factor5	Factor6	Factor7	Factor8
Metribuzin	Triazinone	**0.915**	-0.002	-0.018	0.011	0.005	-0.047	-0.011	-0.004
Metribuzin-diketo	Triazinone	**0.895**	-0.002	-0.016	-0.021	-0.001	0.098	-0.012	0.018

Diuron	Phenylurea	**0.817**	-0.004	-0.029	0.019	0.026	-0.057	-0.006	-0.008
Metribuzin-desamino-diketo	Triazinone	**0.712**	0.007	-0.001	-0.027	-0.007	0.063	-0.011	0.012
CGA 62826	Acylamino acid	**0.520**	0.004	0.129	0.015	-0.027	-0.049	0.055	-0.018
Mecoprop	Phenoxy	0.004	**0.982**	-0.001	-0.001	0.000	0.004	0.000	-0.013
Dichlorprop	Phenoxy	-0.007	**0.976**	0.003	0.000	-0.001	0.004	0.001	0.054
MCPA	Phenoxy	0.005	**0.945**	-0.003	0.001	0.000	-0.004	-0.001	-0.041
Methyl-desphenyl-chloridazon	Pyridazinone	-0.058	-0.002	**0.942**	0.002	-0.001	0.011	-0.009	0.013
Desphenyl chloridazon	Pyridazinone	-0.068	0.001	**0.923**	-0.012	0.013	0.025	-0.015	0.006
CGA 108906	Acylamino acid	0.206	-0.001	**0.788**	0.011	-0.002	-0.032	0.021	-0.014
Simazine	Triazine	-0.010	0.001	0.002	**0.935**	0.003	-0.031	-0.025	-0.020
Simazine, hydroxy	Triazine	0.007	-0.002	-0.001	**0.929**	0.002	0.043	0.050	0.022
Atrazine, desisopropyl	Triazine	-0.012	0.002	-0.003	-0.019	**0.874**	0.038	-0.024	-0.005
DEIA	Triazine	-0.029	-0.002	-0.026	-0.029	**0.655**	0.063	0.026	0.025
Hexazinone	Triazinone	0.009	0.002	0.052	-0.003	**0.562**	0.002	-0.015	-0.050
Atrazine, desethyl-	Triazine	0.035	-0.003	-0.009	0.065	**0.552**	-0.140	0.024	0.030
Desethyl-hydroxy-atrazine	Triazine	0.003	0.001	0.007	-0.170	-0.008	**0.947**	0.065	0.001
Deisopropyl-hydroxyatrazine	Triazine	0.001	0.003	0.000	0.282	-0.004	**0.837**	-0.061	-0.010
Atrazine, hydroxy-	Triazine	-0.008	-0.019	-0.019	0.027	-0.003	0.052	**0.809**	0.031
Didealkyl-hydroxy-atrazine	Triazine	0.016	0.018	0.013	-0.002	0.011	-0.033	**0.799**	-0.035
4-CPP	Phenoxy	0.013	-0.011	0.003	-0.041	-0.020	0.104	-0.024	**0.823**
2-(2,6-dichlorphenoxy) propanoic acid	Phenoxy acid	-0.012	0.011	0.004	0.045	0.019	-0.127	0.021	**0.774**

3.2.3.μg mean, missing = 0

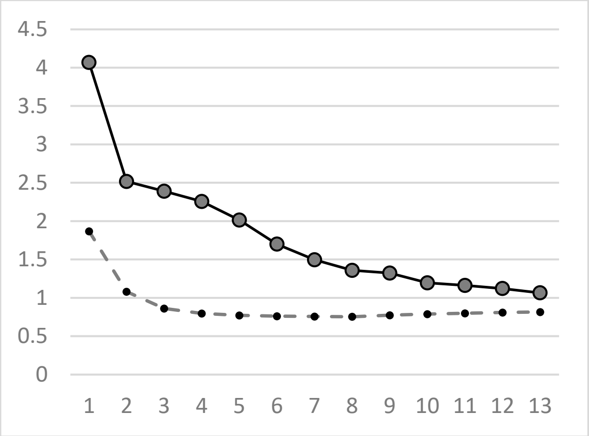

(a)

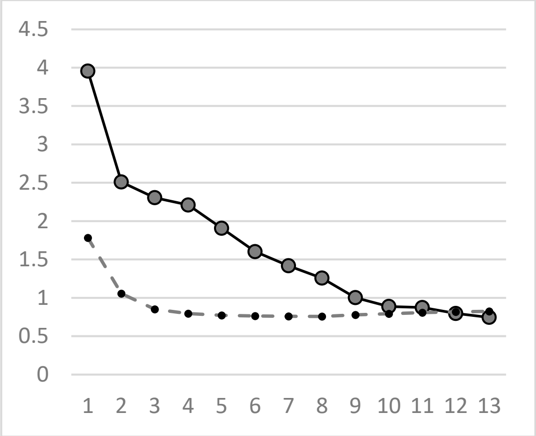

(b)

Figure S14. a) initial scree plot, where 10 factor components were retained, b) final scree plot, where 8 factor components were retained.

The analysis was completed by three iterations.

Table S7. Factor pattern

		Factor 1	Factor2	Factor3	Factor 4	Factor 5	Factor 6	Factor 7	Factor 8
Metribuzin	Triazinone	**0.943**	-0.002	-0.035	-0.067	0.014	0.012	-0.001	0.018
Metribuzin-diketo	Triazinone	**0.929**	0.001	-0.029	0.095	0.010	-0.011	-0.003	-0.011
Diuron	Phenylurea	**0.859**	-0.004	-0.045	-0.064	0.041	0.023	0.000	0.017
Metribuzin-desamino-diketo	Triazinone	**0.842**	0.005	-0.033	0.073	0.003	-0.022	-0.006	-0.021
CGA 62826	Acylamino acid	**0.612**	0.004	0.160	-0.033	-0.056	-0.007	0.006	-0.006
Dichlorprop	Phenoxy	0.001	**0.964**	0.005	-0.019	0.005	0.016	-0.120	-0.009
Mecoprop	Phenoxy	0.001	**0.939**	-0.002	0.055	0.000	-0.007	-0.162	0.074
MCPA	Phenoxy	0.000	**0.699**	-0.004	-0.029	0.005	0.007	**0.535**	-0.067
Methyl-desphenyl-chloridazon	Pyridazinone	-0.081	-0.004	**0.952**	0.014	0.014	0.005	0.000	0.005
Desphenyl chloridazon	Pyridazinone	-0.095	0.005	**0.937**	0.007	0.026	-0.002	-0.006	-0.005
CGA 108906	Acylamino acid	0.385	-0.002	**0.726**	-0.019	-0.035	-0.002	0.010	0.002
Desethyl-hydroxy-atrazine	Triazine	0.003	-0.017	0.002	**0.937**	-0.002	-0.013	0.032	-0.073
Deisopropyl-hydroxyatrazine	Triazine	0.002	-0.013	0.003	**0.922**	-0.002	0.099	0.022	-0.079
2,6-dichlorobenzoic acid	Nitrile herbicides	-0.009	0.054	0.005	**0.652**	-0.019	-0.096	-0.024	0.174
Atrazine, desisopropyl	Triazine	-0.005	0.003	0.009	0.017	**0.887**	-0.013	-0.004	-0.010
DEIA	Triazine	-0.027	-0.020	0.000	0.052	**0.722**	-0.006	0.022	0.094
Atrazine, desethyl-	Triazine	0.037	0.008	0.003	-0.077	**0.557**	0.017	-0.008	-0.040
Atrazine	Triazine	0.013	0.020	0.010	-0.021	**0.529**	0.002	-0.025	-0.066
Simazine	Triazine	-0.035	0.023	0.004	-0.097	-0.004	**0.906**	-0.032	-0.058
Simazine, hydroxy	Triazine	0.034	-0.008	-0.002	0.092	0.005	**0.875**	0.018	0.089
Glyphosate	Organophosphate	-0.003	-0.013	-0.002	-0.016	-0.004	-0.012	**0.915**	-0.004
AMPA	Organophosphate	-0.001	-0.161	0.005	0.052	-0.009	-0.005	**0.581**	0.092
2-(2,6-dichlorphenoxy) propanoic acid	Phenoxy acid	0.003	-0.067	0.000	-0.069	0.011	0.066	0.089	**0.833**

4-CPP	Phenoxy	-0.003	0.101	0.001	0.086	-0.031	-0.037	-0.012	**0.831**

3.2.4.Measurements >QL

Due to the 0/1 data structure the correlation matrix was singular, and therefore not applicable for factor analysis.

3.2.5.Measurements > DWQS

Due to the 0/1 data structure the correlation matrix was singular, and therefore not applicable for factor analysis.
